# The Largest Subunit of RNA Polymerase II as a New Marker Gene to Study Assemblages of Arbuscular Mycorrhizal Fungi in the Field

**DOI:** 10.1371/journal.pone.0107783

**Published:** 2014-10-02

**Authors:** Herbert Stockinger, Marine Peyret-Guzzon, Sally Koegel, Marie-Lara Bouffaud, Dirk Redecker

**Affiliations:** 1 Université de Bourgogne, UMR1347 Agroécologie, Dijon, France; 2 INRA, UMR1347 Agroécologie, Dijon, France; 3 Botanical Institute, Basel, Switzerland; University of Tartu, Estonia

## Abstract

Due to the potential of arbuscular mycorrhizal fungi (AMF, *Glomeromycota*) to improve plant growth and soil quality, the influence of agricultural practice on their diversity continues to be an important research question. Up to now studies of community diversity in AMF have exclusively been based on nuclear ribosomal gene regions, which in AMF show high intra-organism polymorphism, seriously complicating interpretation of these data. We designed specific PCR primers for 454 sequencing of a region of the largest subunit of RNA polymerase II gene, and established a new reference dataset comprising all major AMF lineages. This gene is known to be monomorphic within fungal isolates but shows an excellent barcode gap between species. We designed a primer set to amplify all known lineages of AMF and demonstrated its applicability in combination with high-throughput sequencing in a long-term tillage experiment. The PCR primers showed a specificity of 99.94% for glomeromycotan sequences. We found evidence of significant shifts of the AMF communities caused by soil management and showed that tillage effects on different AMF taxa are clearly more complex than previously thought. The high resolving power of high-throughput sequencing highlights the need for quantitative measurements to efficiently detect these effects.

## Introduction

Arbuscular mycorrhiza is an extremely widespread mutualistic symbiosis between plants and obligatory symbiotic fungi from the phylum *Glomeromycota*. About 240 species of glomeromycotan fungi have been described so far, based on their spore morphology and molecular phylogenetic data [1], [2]. Most crop plants are hosts to this association and crop production may potentially benefit from it [3]. Therefore the relationship between different types of agricultural production on one hand and assemblages and populations of arbuscular mycorrhizal fungi (AMF) on the other hand has been in the focus of research activity.

Traditionally, AMF have been identified based on their spore morphology. As only few morphological characters are present, this is not a trivial task, in particular when using field-collected spores which may lack crucial features due to degradation caused by other soil biota. Spores of very similar morphologies are now known to be produced by phylogenetically distant AMF [4]. Several biases are implicit to this approach, namely the overestimation of the importance of prolific sporulators, and on the other side the non-detectability of others that scarcely form spores. Moreover, spores represent the inoculum potential of the soil, not necessarily the symbiotically active population. To avoid these potential problems, molecular methods have been developed which allow the detection of AMF in soil as well as within colonized roots, allowing groundbreaking insights into AMF ecology, e.g. the study by Helgason *et al.* [5]. Since then, numerous field studies on AM fungal ecology have been conducted using molecular markers (e.g. [6], [7], [8]). However, the only genes used in studies on species-level diversity were different regions of nuclear-encoded ribosomal RNA, which in the *Glomeromycota* show numerous slightly different variants within the fungal organism. This fact complicates the separation of closely related species and annotation in large-scale diversity surveys, such as deep sequencing approaches, which offer superior depth of analysis than cloning-sequencing approaches. In fact, previous studies have shown that considerable parts of fungal diversity may remain unfathomed due to technical constraints inherent to cloning and sequencing techniques [8].

In phylogenetic studies, the need to study several loci in order to improve reliability and resolution power of phylogenies is widely recognized [9], [10], therefore several alternative marker genes have been proposed in this context also for the *Glomeromycota*. The largest subunit of RNA polymerase II (RPB1) gene stood out for several reasons: it has several variable regions useful for species discrimination; according to present knowledge it is a single-copy gene in fungi, avoiding problems with paralogues. In fact, the genome sequence of *Rhizophagus irregularis* [11] shows no intra-isolate variants of this gene. These properties render this marker very attractive for large-scale diversity surveys of the *Glomeromycota*. In the present study, we therefore assembled a reference database of RPB1 sequences and designed primers for the *Glomeromycota* to address the possible use of this marker gene for field studies of AMF.

We tested the usefulness of the primers for high-throughput sequencing for the analysis of species assemblages of AMF in maize roots in different tillage treatments in a long-term experimental site in Tänikon, Switzerland. This site has been previously analyzed using different spore-based and molecular approaches at the species and intraspecies level [12], [13], [14], providing us the advantage of relating our results to a large body of findings. Tillage has been suggested to be “the most unique and strongest agricultural selection pressure for mycorrhizal symbionts” [15]. Others studies in different locations have used DNA fingerprinting methods or cloning/sequencing approaches [5], [16], [17], thus to our knowledge this is the first study addressing tillage effects on AMF communities in roots in arable soils by using 454 sequencing.

## Materials and Methods

### Fungal reference cultures

Spores of most AMF species of the reference dataset were obtained from the ‘International Bank for the *Glomeromycota*’ (BEG/IBG) at Dijon (France) or from the ‘International culture collection of (vesicular) arbuscular mycorrhizal fungi’ (INVAM, Morgantown, USA). For details see Table 1. The recently proposed nomenclature of the *Glomeromycota* of Redecker *et al.* [2] is used. The authors would like to thank Joe Morton (INVAM) and Valérie Monfort-Pimet (BEG) for providing fungal cultures.

**Table 1:** Biological materials, Primers and PCR conditions used for the reference datasets.

Accession	Name	Isolate, origin
HG315978	*Acaulospora laevis*	BEG242/LPA47, IBG
HG315982	*Acaulospora laevis*	BEG242/LPA47, IBG
HG315975	*Acaulospora longula*	BEG8/LPA46, IBG
HG316014	*Ambispora leptoticha*	CR312, INVAM
HG316019	*Ambispora leptoticha*	CR312, INVAM
HG315988	*Archaeospora trappei*	CR401B, INVAM
HG316013	*Cetraspora nodosa*	BEG4/LPA50, IBG
HG316018	*Claroideoglomus claroideum*	BEG23/LPA31, IBG
HG315991	*Claroideoglomus etunicatum*	BEG247/LPA61, IBG
HG316016	*Dentiscutata erythropus*	HA150B, isolate from INVAM, cultivated at IBG
HG316017	*Dentiscutata erythropus*	HA150B, isolate from INVAM, cultivated at IBG
HG316012	*Dentiscutata heterogama*	BEG35/LPA39, IBG
HG315981	*Diversispora epigea*	BEG47/LPA10, IBG
HG315973	*Funneliformis mosseae*	BEG246/LPA58, IBG
HG315993	*Funneliformis sp.2*	trap culture (AGc)
HG315994	*Funneliformis sp.2*	trap culture (AGc)
HG315995	*Funneliformis sp.2*	trap culture (AGc)
HG315998	*Gigaspora candida*	BEG17/LPA52/HE-MOS, IBG
HG316011	*Gigaspora margarita*	BEG34/LPA2, IBG
HG315979	*Gigaspora rosea*	BEG9/LPA23, IBG
HG315980	*Gigaspora rosea*	BEG9/LPA23, IBG
HG315989	*Glomus cf. diaphanum*	BR608b-3, INVAM
HG316006	*Glomus macrocarpum*	Att1495-16, Chris Walker collection
HG316007	*Glomus macrocarpum*	Att1495-16, Chris Walker collection
HG316021	*Glomus macrocarpum*	Att1519-0, Chris Walker collection
HG315968	*Glomus sp.* MES-190	field collection, MES-190, Argentina, leg. Matthew E.Smith
HG315969	*Glomus sp.* MES-190	field collection, MES-190, Argentina, leg. Matthew E.Smith
HG315974	*Paraglomus brasilianum*	BEG 239/LPA25, IBG
HG315976	*Paraglomus brasilianum*	BEG239/LPA25, IBG
HG315977	*Paraglomus brasilianum*	BEG239/LPA25, IBG
HG316010	*Paraglomus occultum*	CL700A, INVAM
HG315985	*Paraglomus sp.*	root sample, long-term experimental field site, INRA Lusignan, France
HG315986	*Paraglomus sp.*	root sample, long-term experimental field site, INRA Lusignan, France
HG315997	*Racocetra castanea*	BEG1/LPA4, IBG
HG316003	*Racocetra cf. weresubiae*	trap culture
HG315970	*Redeckera fulva*	field collection CL/MART06.056, Martinique, leg. Claude Lecuru, det. Dirk Redecker, herbarium Université de Lille
HG315971	*Redeckera fulva*	field collection CL/MART06.003, Martinique, leg. Claude Lecuru, det. Dirk Redecker, herbarium Université de Lille
HG315972	*Redeckera fulva*	field collection CL/MART06.057, Martinique, leg. Claude Lecuru, det. Dirk Redecker, herbarium Université de Lille
HG316001	*Rhizophagus clarus*	BEG248/LPA64, IBG
HG316004	*Rhizophagus clarus*	WV235, INVAM
HG316024	*Rhizophagus clarus*	BEG142/LPA16, IBG
HG316020	*Rhizophagus intraradices*	MUCL49413, root organ culture, GINCO, Belgium
HG315983	*Rhizophagus irregularis*	LPA71/DAOM197198, cultivated at IBG
HG315984	*Rhizophagus irregularis*	LPA71/DAOM197198, cultivated at IBG
HG315987	*Rhizophagus irregularis*	BEG141/LPA8, IBG
HG315992	*Rhizophagus irregularis*	MB002, isolate from Austria, Stockinger collection
HG315996	*Rhizophagus irregularis*	BEG145, Stockinger collection
HG315999	*Rhizophagus irregularis*	JJ141, root organ culture established by J. Jansa, Switzerland
HG316000	*Rhizophagus irregularis*	JJ746, root organ culture established by J. Jansa, Switzerland
HG316002	*Rhizophagus irregularis*	BEG244/LPA54, IBG
HG316008	*Rhizophagus irregularis*	BEG144, IBG
HG316009	*Rhizophagus irregularis*	BEG235/LPA7, IBG
HG316015	*Rhizophagus irregularis*	JJ746, root organ culture established by J. Jansa, Switzerland
HG316022	*Sclerocystis cf. pubescens* HS-2013	environmental sample, Edinburgh, Scotland, leg. et det. Chris Walker
HG316023	*Sclerocystis cf. pubescens* HS-2013	environmental sample, Edinburgh, Scotland, leg. et det. Chris Walker
HG315990	*Sclerocystis sinuosa*	MD126, INVAM
HG316005	*Septoglomus viscosum*	U1, University of Alessandria, Italy

IBG: International Bank for the Glomeromycota (Dijon, France), INVAM: International Collection of Vesicular-arbuscular and Arbuscular Mycorrhizal Fungi (West Virginia University, Morgantown, USA).

### DNA Extraction

DNA of single or multiple spores was extracted according to Naumann *et al.* [18] with 5–10 µl of 5× GoTaq Flexi Reaction Buffer (Promega, Fitchburg, USA) or 10× MP Taq Pol buffer without MgCl_2_ (MP Biomedicals, Santa Ana, USA) (see Table S1). In some cases, the DNeasy plant mini kit (Qiagen, Hilden, Germany) was used to extract DNA of a larger quantity of spores (see Table S1).

### PCR reference dataset and Sanger sequencing

**Figure 1:**
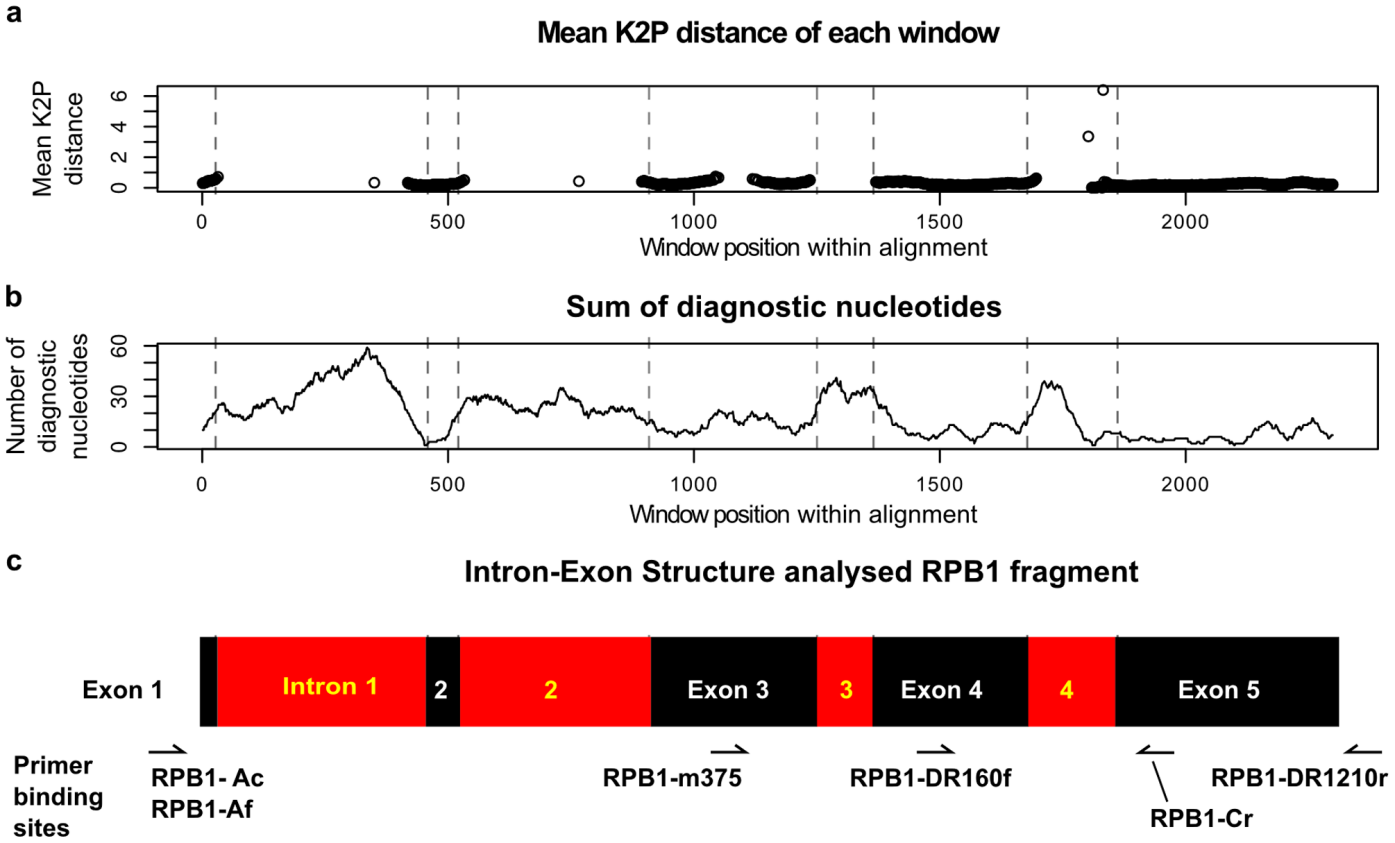
Analysis of structure and variability of partial RPB1 genes in the longer reference dataset 1. A 50-bp sliding window was used to generate (a) and (b). (a): Mean K2P distances of pairwise comparisons of the sequences: the mean K2P distances within the introns are higher than 6%, therefore they are outside the range of the x-axis. Consequently, the black lines indicate that the exons have a relatively low mean K2P distance. (b): the sum of diagnostic nucleotides along the alignment as analysed using SpideR. A higher number of diagnostic nucleotides indicate good separation of species. (c): corresponding intron-exon structure of partial RPB1, including primer binding sites within the alignment.

Nested PCRs were performed with Phusion High Fidelity DNA polymerase (Finnzymes, Vantaa, Finland). Several new primers were designed and used in combination with published primers (Table S1, Figure 1). The 1^st^ PCR was performed with 0.02 U/µl Phusion polymerase, 1 µl genomic DNA, 1× Phusion HF Buffer, 0.5 µM of each primer, 0.2 mM of each dNTPs in a total volume of 20 µl. The same setup was used for the 2^nd^ round of the nested PCR, for which 0.5 µl of the first PCR was used as template. Cycling conditions were adjusted according to the primer combination used. Primers with corresponding PCR cycling conditions are listed in Table S1. PCR products were cloned using the StrataClone Blunt PCR Cloning Kit (Agilent, Santa Clara, USA) according to manufacturer's protocol. Several clones were amplified by PCR with vector primer and clones with the correct insert where selected for further analyses. Clones were grown overnight in liquid Terrific Broth medium and plasmids were purified using a NucleoSpin Plasmid Kit (Macherey-Nagel, Düren, Germany). Sanger sequencing was performed by GATC (Konstanz, Germany) or Eurofins MWG Operon (Ebersberg, Germany). Sequences were checked and assembled using the Staden package [19]. All sequences were aligned in ARB [20]. Intron-exon structure was determined in silico by comparison with published RPB1 gene structures and with the amino acid sequences. The sequences were submitted to the EMBL database under the accession numbers HG315968-HG316024 (see Table 1).

### RPB1 reference dataset 1

**Figure 2:**
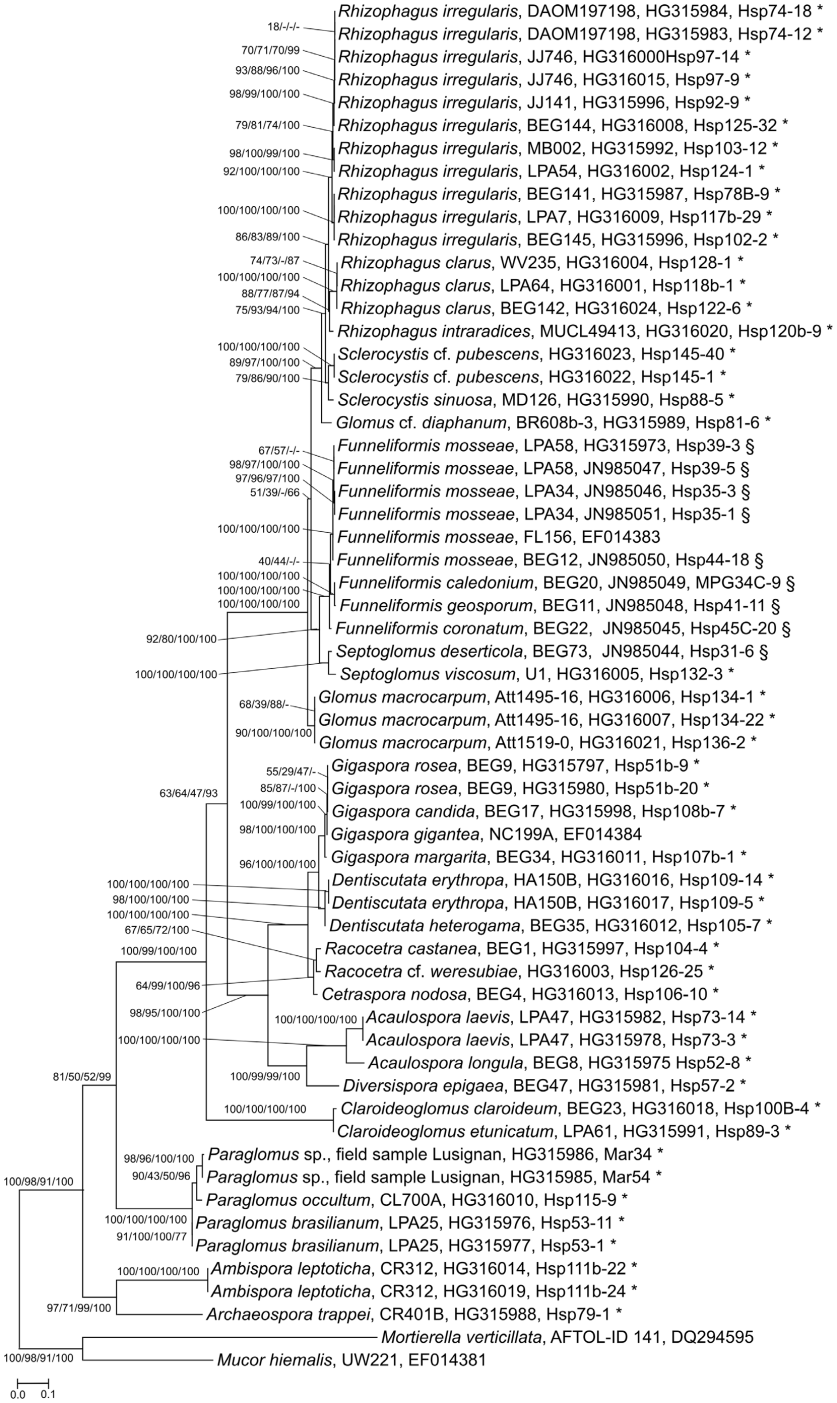
RAxML phylogenetic tree based on the exon of the fragment RPB1-Ac and RPB1-DR1210. Support values are from RAxML (1000 bootstrap replicates (BR)), MP (1000 BR), NJ (1000 BR) and MrBayes. * Sequences from this study, § Sequences contributed to Schoch et al. [39].

This first dataset comprises a fragment of about 1.5–2.1 kb length and was initially used for primer design, phylogenetic analysis and for identifying variable regions. Around 1233 sites of the aligned exon sequences, were used for constructing phylogenetic trees with RAxML [21] (Figure 2), MEGA5 [22] and MRBAYES [23]. The fragment was additionally analysed for variable regions with the package SPIDER [24] of R version 2.15.1 [25].

### RPB1 reference dataset 2

This dataset contains the abovementioned long sequences and additional shorter sequences spanning the primers RPB1-DR160f to RPB1-DR1210r (Table S1). This increased the number of comparable sequences for 454 sequence analysis. Barcode gap and variability analyses were performed on this fragment using SPIDER.

The aligned sequences were used as references for 454 data analysis, first as reference alignment and after performing 100 inferences of ML search with RAxML [21] as reference tree for EPA RAxML analysis [26].

### Field site and sampling

The sampling field site was part of a long-term tillage experiment in Tänikon (Switzerland, (47.4862 N, 8.9195 E)). The site was established in 1987. Details of the experimental setup and soil properties are described by Anken *et al.* [27] and Jansa *et al.* [12], [13]. In short, the experiment had six different treatments and was organized in a randomized block design. Each treatment was replicated four times. The site is subjected to crop rotation and *Zea mays* was the crop growing at sampling time in June 2007. Samples were taken from three different treatments: no-tillage (maize was planted directly into the soil), chisel (soil was loosened with a share chisel to a depth of 25 cm without turning the soil) and tillage (soil was ploughed to a depth of 25 cm and turned around). Four plants per plot from each of the four replicate plots of each treatment were collected, a total of 48 plants (Figure S1). Several aliquots of 50–80 mg (fresh weight) root fragments per sample were frozen in liquid nitrogen and stored at −80°C. Details of the sampling procedure were described by Börstler *et al.* [14]. We thank Jan Jansa (ETH Zürich) and Thomas Anken (Agroscope Reckenholz-Tänikon) for providing access to the field site and Boris Börstler, Zuzana Sýkorová and Odile Thiéry for sampling.

### DNA extraction, PCR and pyrosequencing

DNA of roots was extracted using a DNeasy plant mini kit (Qiagen, Hilden, Germany). The roots were ground in liquid nitrogen and DNA was extracted according to the manufacturer's protocol. The DNA was eluted twice with 70 µl and 50 µl buffer AE. For two samples, the same DNA extract used by Börstler *et al.* [14] from this sampling was reused instead, because no root aliquots were left (one sample 4 (47-4) from the chisel treatment and one from the no tillage treatment 2 (15-1)).

The 1^st^ PCR of the nested reaction was performed using 0.02 U/µl Phusion polymerase, 1 µl genomic DNA, 1× Phusion HF Buffer, 0.5 µM of each primer (RPB1-HSm375-mix7 and RPB1-1210+linker, Table S2), 0.2 mM of each dNTPs, 0.4 µg/µl BSA, 3% DMSO in a total volume of 20 µl. The cycling parameters were: 30 s at 98°C, 35 cycles of 10 s at 98°C, 30 s at 60°C and 30 s at 72°C, followed by 10 min at 72°C (Eppendorf Mastercycler epgradient S, Vaudaux-Eppendorf, Schönenbuch, Switzerland). Each DNA extract was amplified in three replicates and amplification success was checked on an agarose gel. The three replicates were pooled and purified using the Zymo Research DNA Clean & Concentrator - 5 Kit (Zymo Research Corporation, Irvine, USA). The PCR product was eluted in 20 µl of sterile water.

In the nested PCR-approach, the adapters for 454 sequencing and barcodes were added in the second round as previously suggested [28]. In addition, this approach made it possible to sequence directionally from the primers containing the TitA adaptor. Barcodes of 9 bp with 4 base differences between each other were selected with the software BARCRAWL [29] and are listed in Table S2 (see also Fig. S2). The nested PCR was performed with 0.02 U/µl Phusion polymerase, 1 µl purified PCR product, 1× Phusion HF Buffer, 0.5 µM of each primer (TitB-adaptor+RPB1-DR160f-mix10 and linker+Barcode+TitA-adaptor, Table S2), 0.2 mM of each dNTPs, 0.4 µg/µl BSA, 3% DMSO in a total volume of 20 µl. Cycling parameters were 30 s at 98°c, 10 cycles of 10 s at 98°C, 30 s at 64°C, 30 s at 72°C, followed by 10 min at 72°C (Eppendorf Mastercycler epgradient S). Each purified PCR product from the first round was again amplified three times and products were pooled. PCR products were verified on an agarose gel.

The PCR product of the nested approach was run on a 1.5% agarose gel (1% SeaKem-0.5% NuSieve; Cambrex Bio Science, Rockland, USA) and bands within the range of 650 bp–1 kb were cut out, mainly to remove fragment from the first PCR. The DNA fragments were recovered from the gel in 20 µl of water according to the Zymoclean Gel DNA Recovery Kit protocol (Zymo Research Corporation, Irvine, USA). Recovered DNA fragments were verified visually on a 1% agarose gel.

Each of the four sub-samples of a plot were pooled and the concentration, size and quality of the pooled samples was analysed with the Bioanalyzer 2100 (Agilent, Santa Clara, USA). All samples were mixed equimolarly for the final 454 PCR amplicon sample, which was sent to GATC (Konstanz, Germany) for sequencing 6×1/16 regions of a 454 GS FLX+ plate. The whole data set was submitted to Sequence Read Archive (ERP002624).

### 454 sequence editing and analysis

Sequences obtained from the different 1/16 regions (lanes) were pooled to maximize coverage. The data were analysed mainly with the QIIME pipeline [30] with some modifications based on the protocol of Yu *et al.* [31]. For details of the workflow overview, see Data File S1 and Figure S3.

Primer, linker and barcode sequences were removed from raw 454 data and split into the different libraries depending on the barcode. Sequences were filtered out if they were shorter than 200 bp or had an ambiguous nucleotide. Using PyNAST [30], sequences were aligned with the reference alignment at a minimum similarity of 60%. Sequences which failed to align were discarded. Then the sequences were clustered at 99.7% similarity with USEARCH [32], which increased the speed of downstream processing. *De novo* chimera detection was performed using UCHIME in USEARCH [33]. MACSE [34] was used to denoise sequences. MACSE allows to take advantage of protein-coding genes (such as RPB1), aligning sequences at the amino acid level to reference sequences and searching for stop codons and frame-shifts caused by homopolymer errors. OTU picking at 99.2% similarity was performed using CROP [35]. The standard QIIME method was used to assign taxonomic information to each OTU using reference dataset 2. In addition, the taxonomic assignment was verified by two other methods. Firstly, the EPA of RAxML [25] (Figure S4) was used and secondly a bootstrapped Neighbor Joining tree based on K2P distances (Figure S5, 1000× bootstrap replicates) was constructed using MEGA5 [22]. The taxonomic assignment was verified with the latter two analyses and a consensus assignment used for further analyses. QIIME assignments were compared with EPA-RAxML and NJ analysis. If the QIIME assignment did not match or could be refined by the other two analyses, the tree-based assignment was preferred. EPA-RAxML was valued higher than NJ analysis, because it is specially developed to assign shorter sequences to a reference tree. If the three assignment methods were in disagreement, the assignment was set to a common taxonomic sublevel. In a final step, OTUs were checked for singletons, chimeric and non-glomeromycotan sequences. These sequences were excluded from further analysis.

### Community diversity analysis

For further analysis, sequences of the OTUs were aligned and a phylogenetic tree was constructed in QIIME. This tree served as the base for beta diversity and alpha diversity analyses. For further analyses, samples were normalized through equal sampling depth of 1400 sequences per sample. Rarefaction ananlyses were performed within QIIME, based on OTUs per number of sequences. As a measure of beta-diversity, the number of reads for each OTU/total reads per sample was used. Unweighted and weighted Unifrac [36] matrices were calculated and used for further analysis. Analysis of similarities (ANOSIM) was used to detect significant differences between the treatments. In addition, the treatments tillage and chisel were grouped and compared with the no tillage treatment. ANOSIM analyses were performed with 2000 permutations within QIIME and mothur [37], because mothur shows which treatments are significantly different. We visualized the dissimilarity matrix in a Principal Coordinates Analysis (PCoA) and analyzed the variability of the distances within and between treatments (QIIME). Results

### RPB1 as a marker gene in the *Glomeromycota*

Reference dataset 1, based on 1.5–2 kb fragments of the RPB1 gene, was used for primer design and contained a total of 30 cultured and named species and in addition two environmental sequences. Sequences of nine of these species were either partially or entirely published previously by other authors [9], [38], [39]. For *Rhizophagus irregularis*, *Paraglomus occultum* and *Dentiscutata heterogama*, we could amplify this whole long fragment of RPB1 and used it for further analysis. Almost all major lineages of glomeromycotan fungi are covered, from *Paraglomus* to *Rhizophagus*, including the type species for the *Glomeromycota*, *Glomus macrocarpum* (Figure 2).

In order to estimate intra-isolate variation of RPB1 in the *Glomeromycota*, we sequenced two (in some cases three) clones of ten isolates from a broad range of different lineages across this phylum (Figure 2), including multiple isolates of *Rh. irregularis* and *Fu. mosseae*. In no case did we detect a variation of more than 0.7% over 2138 bp (*Am. leptoticha*). Intra-isolate variation between two clones each of *Fu. mosseae* isolates LPA34 and LPA58, for instance, was 0 to 0.05%, introns included, whereas the sequences of the two isolates differed by 1.24%. We reanalyzed some previously-published RPB1 sequences of *Rh. proliferus* [38]: Four clones obtained by amplification using Taq polymerase (which has a higher error rate than the proof-reading enzymes we used in the present study; accession numbers AM284979.1 to AM284982.1) showed a similarity exceeding 99% over 1534 bp.

Figure 1 shows that the mean K2P distance among taxa and the number of diagnostic nucleotides are the highest in the first intron. Among the exons, exon 3 and the analysed part of exon 5 showed the highest number of diagnostic nucleotides.

**Figure 3:**
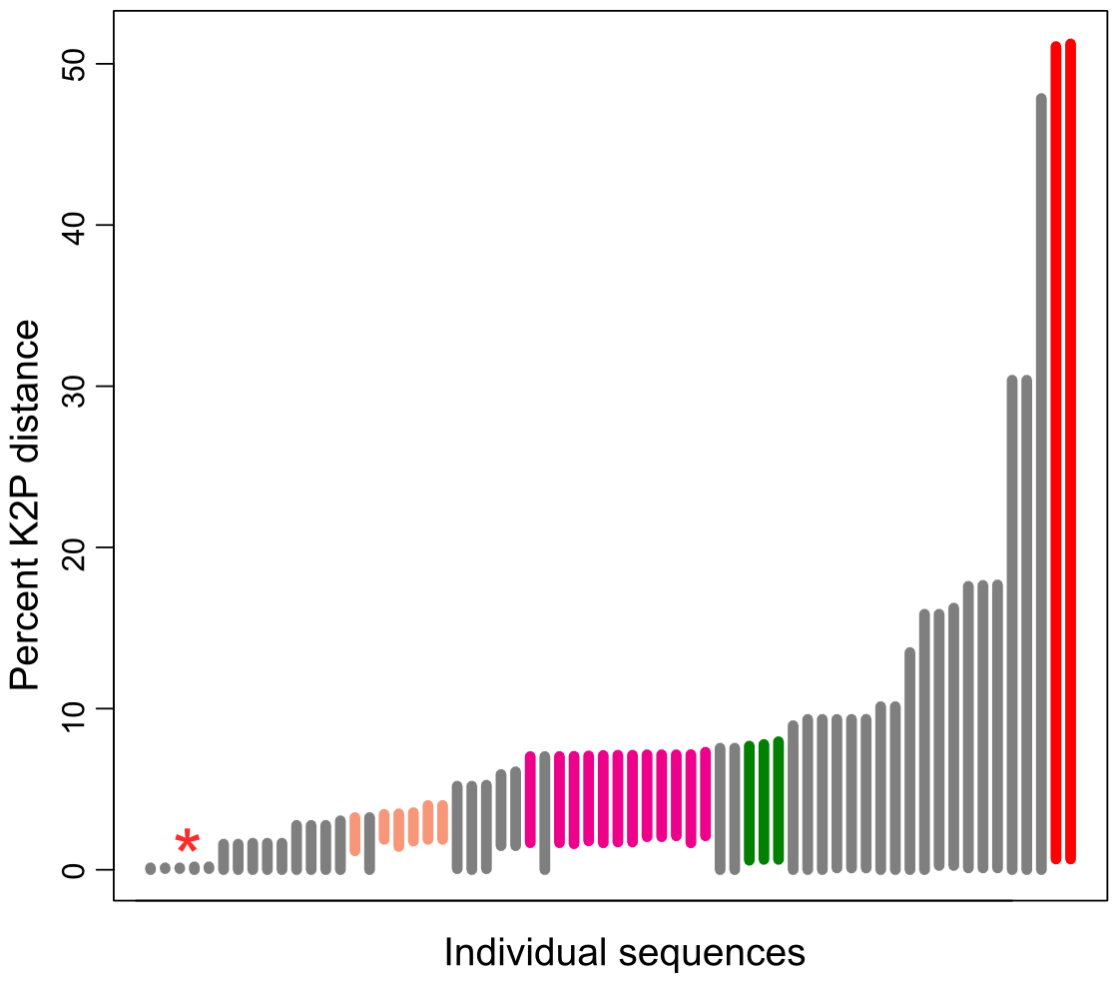
Barcode gap analysis of the RPB1 fragment RPB1-Ac to RPB1-DR1210r, based on K2P distances. Every sequence was plotted with its corresponding intraspecific variation and minimum interspecific variation. Bars indicate K2P difference between the maximum intraspecific (bottom) and minimum interspecific divergence (top) of each sequence. The red asterisk indicates sequences with small K2P distances of the three species *Gigaspora rosea*, *Gi. gigantea* and *Gi. candida*, which are not resolved. Two of these species are assumed to be conspecific. Colored bars represent sequences of the following species: apricot - *Funneliformis mosseae*, magenta – *Rhizophagus irregularis*, green – *Rhizophagus clarus*, red – *Ambispora leptoticha*. Bars touching zero at their lower end stand for species represented by a single sequence.

Barcode gap analysis (Figure 3) in all cases detected a gap between the largest intraspecific and the smallest interspecific variation based on the K2P distance. However, five sequences of the species of *Gigaspora rosea*, *Gi. candida* and *Gi. gigantea* have an extremely small gap of 0.1–0.2% K2P distance. The maximum intraspecific variation reaches a maximum of 2.1% K2P distances in *Rh. irregularis sensu lato*.

RPB1 reference dataset 2 was used for the 454 sequence analysis. It contained shorter sequences of 34 named species and four environmental clusters, spanning at least the primer pairs RPB1-DR160f and RPB1-DR1210r. Barcode gap analysis showed similar results as the longer fragment in dataset 1, and equally no sequence variation among the three species *Gigaspora rosea*, *Gi. gigantea* and *Gi. candida* (Figure S6). All other groups showed a gap of at least 0.9% K2P distance. Based on these data, we set 0.8% as threshold for defining OTUs in the 454 data analysis.

### 454 Community analysis

#### Sequences/OTUs

After removing sequences shorter than 200 bp including primers, barcode and adapters, 23263 sequences were available for further analysis. Without primers, barcode and adapter, the sequence length range was between 145 and 515 bp, with a mean of 254 bp, whereas half of all sequences had a length of 185 to 328 bp.

After removing chimeric, non-AMF sequences and singletons, 22776 sequences were analysed. Overall, only 15 sequences of non-AMF origin were found in the dataset, corresponding to 0.06%. Sequence numbers per sample ranged between 1425 and 2770 sequences. The clustering generated 105 OTUs based on a 0.8% cut-off level.

The rarefaction analysis of each sample based on observed OTUs indicated the sampling was sufficient to obtain a great majority of AMF OTUs (Figure S7). The total number of OTUs in each sample varies from 15 to 55 OTUs. Interestingly, both the highest and the lowest values were from no-tillage treatment samples. The four combined samples of the no-tillage treatment contained 76 OTUs, followed by the tillage and the chisel treatments, with 67 and 59 OTUs, respectively (Tab. S3).

#### Community analysis based on UniFrac distance

**Figure 4:**
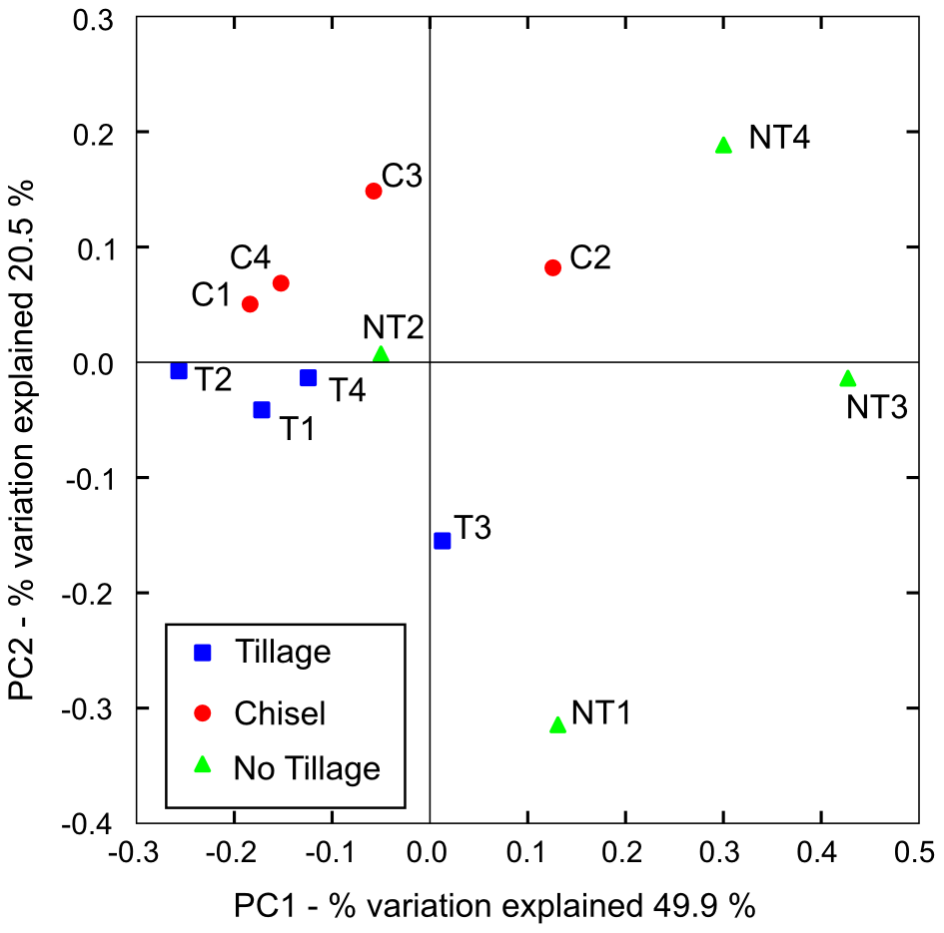
Principal Coordinates Analysis of weighted UniFrac distances.

The PCoA (Principal Coordinates Analysis) based on weighted UniFrac distances shows a tendency of the disturbed sites (tillage/chisel) to separate from most non-till plots (Figure 4). Weighted PCoA also revealed some clustering of the tillage and chisel treatments, whereas the samples of the no-tillage treatment did not form any cluster, highlighting the heterogeneity of the different plots of this treatment (Figure S8).

To validate the clustering we used ANOSIM (analysis of similarities) to analyse the different groupings. When comparing the three different treatments, the weighted UniFrac distances ANOSIM was significant at a p-value of <0.05. Nevertheless, the R-value of 0.30 suggests a moderate separation of these three groups.

#### Taxon identification & distribution

Because of length differences of the fragments and the relatively small reference dataset, we applied three different mapping methods: the QIIME RDP algorithm, EPA- RAXML and a NJ K2P distance tree. All three methods revealed that some species contain several OTUs. This is particularly obvious in the two well-sampled species *Rh. irregularis s. l.* (one of which corresponded to the BEG141 clade, see below) and *Fu. mosseae*. Indeed, 14 OTUs were identified as *Rh. irregularis* and six in the case of *Fu. mosseae*. On the other hand, other species were represented just by one OTU, such as *Ce. nodosa*.

**Figure 5:**
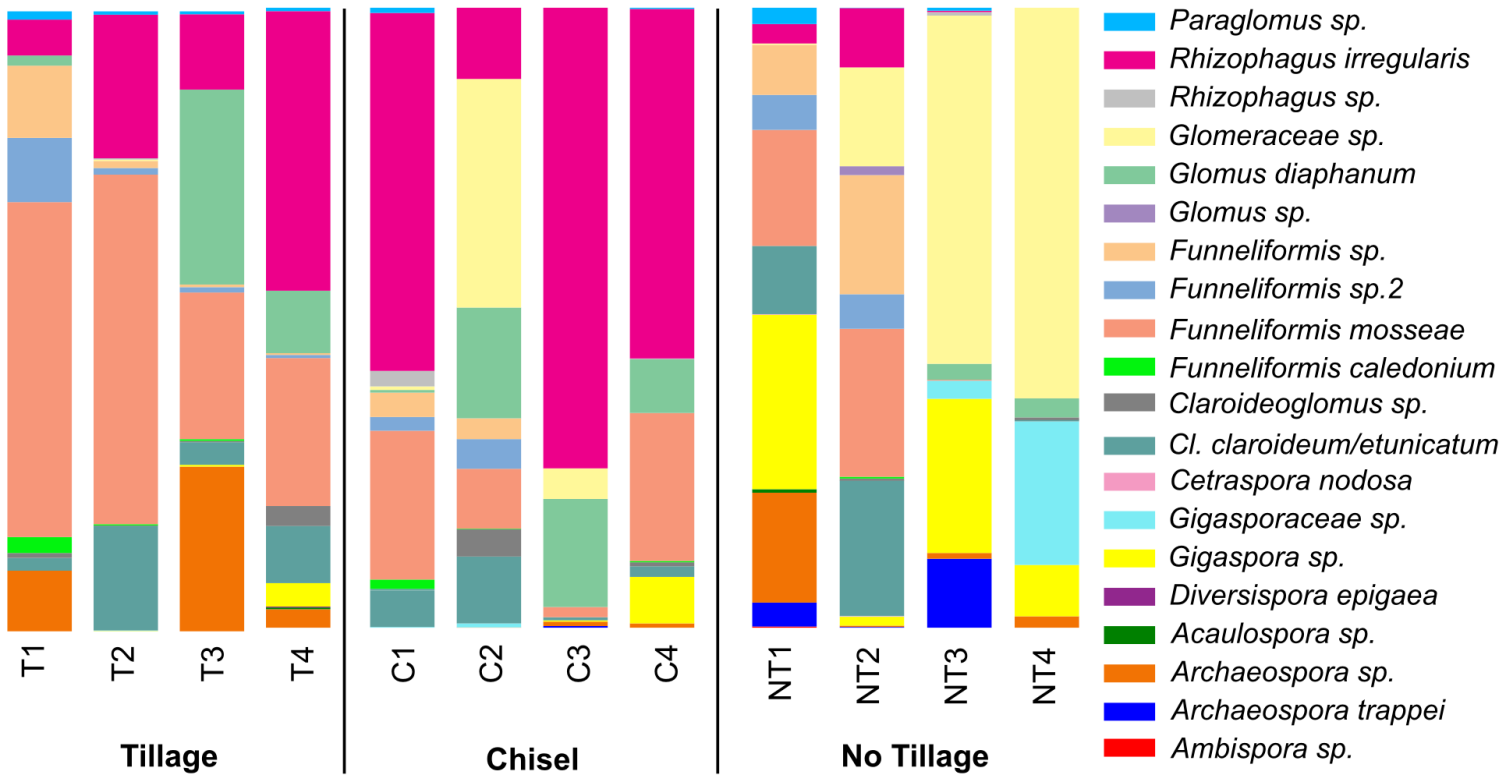
Relative phylogenetic taxon distribution in the different treatments and plots.

Overall, we identified seven environmental molecular taxa (MTs) to known species: *Ar. trappei*, *Di. epigea*, *Ce. nodosa*, *Fu. caledonium*, *Fu. mosseae*, *Gl. cf. diaphanum* and *Rh. irregularis* (Figure 5 & S9). Although our *Cl. claroideum* and *Cl. etunicatum* isolates could be clearly distinguished using rpb1 sequences, many sequences from the field were too short and lacked the diagnostic sites. Therefore we defined *Cl. etunicatum/Cl. claroideum* as one MT, distinguishing it from other, more deeply divergent *Claroideoglomus* lineages without close known relatives. The environmental molecular taxa *Glomeraceae sp.* and *Gigasporaceae* sp. had no close matches above the family level. In total, we combined the 105 OTUs to 20 species-level MTs.

In the no-tillage treatment, 19 MTs were detected, that is, all were occurring except *Di. epigea*. In the tillage treatment 16 MTs were found, 15 in the chisel treatment. The chisel and the tillage treatments were dominated by *Rh. irregularis* and by *Fu. mosseae*, respectively. These two species were strongly reduced in sequence number in the no-tillage treatment (Figure S9). In contrast, the sequence number of *Gigaspora* sp., *Glomeraceae* sp, and *Archaeospora trappei* increased in the no-tillage treatment (Figure S9). The molecular taxon *Glomeraceae* sp. dominated the no-tillage treatment. Out of 20 MTs, only five were not found in all three treatments. Four of these five were rare everywhere (that is, represented by two to 20 sequences in the experiment), only *Ar. trappei* was present in substantial amounts in the no-tillage treatment (212 sequences).

## Discussion

RBP1 was previously identified as a highly useful marker gene for phylogenetic analyses in addition to ribosomal genes [9] in fungi in general, as well as in the *Glomeromycota* [38]. It was also tested as an alternative maker for DNA barcoding of fungi [39]. Although RPB1 performed superior in that study when compared to the official DNA barcode ITS with regard to species resolving power, it was not selected as fungal DNA barcode. Indeed, one of the reasons of this preference was a considerably lower global PCR amplification success rate of RPB1 due to the lack of appropriate primers for many groups of fungi [39]. In addition, the number of sequenced species in that study was relatively limited in fungi, particularly in AMF [38], [39]. In this study, we improved the taxon sampling of RPB1 sequences in the *Glomeromycota* by providing a reference dataset of 56 sequences comprising all major lineages, based on sequences from spores from well-characterized cultures. Moreover, we designed new primers targeting subgroups of the *Glomeromycota* based on this dataset, which was essential to PCR success in this study. The new primers generated products from all samples from the Tänikon site, while at the same time they showed a specificity of 99.94%, which is by far superior to previous approaches using different marker genes. Depending on the study and the primers used, about 50–70% of the sequences of nuclear ribosomal genes were reported to be of non-glomeromycotan origin [6], [17], [40].

Nuclear ribosomal LSU and ITS regions are present in numerous tandem repeats within a nucleus. The variation within the ITS region of one spore and species can be up to 20% [41], which is among the highest levels of variability reported in the fungal kingdom [42]. RPB1 is, to current knowledge, a single copy gene in fungi [39] and all available data, including the genome sequence of *Rh. irregularis* DAOM 181602, indicate that it is monomorphic within a given AMF isolate. This fact will greatly simplify the analysis of sequence data from high-throughput sequencing analyses where numerous slightly different nuclear rDNA variants recovered from the same species are difficult to interpret. On the other hand, PCR problems that could be expected due to low copy number of RPB1 in the glomeromycotan genome were not encountered, as amplicons were obtained from all root samples [43], [44]. Our data on *Rh. irregularis* and *Fu. mosseae* isolates indicate that there is some polymorphism among isolates, which (in contrast to nuclear ribosomal gene regions with high intragenome polymorphism) could potentially be taken into account for estimates of genetic diversity in the field. However this may be currently difficult due to sequencing error rates. Inter-isolate variation in *Rh. irregularis* in the long fragment of RPB1, including the highly variable introns, was up to 2.1% of K2P distance. In fact, one of the introns was previously used to separate intraspecific genotypes of *Rh. irregularis* [45], [46]. Intraspecific variation was considerably lower in the exons alone. On the other hand, the three isolates of *Rhizophagus clarus* we analyzed showed only very weak sequence divergence (0.6% K2P distance).

The high intraspecific variation reaching a maximum of 2.1% K2P distances in *Rh. irregularis sensu lato* is partly attributable to the genetic distance between two closely-related but distinct clades, exemplified by the isolates DAOM 197198 and BEG141, respectively. Using mitochondrial rDNA sequences, it was previously shown that the BEG141 clade is well-separated (haplotypes XXII, XXIII and XXV in Börstler *et al.* [14]) and it can be speculated that it represents a species different from *Rh. irregularis*. Defining these two groups as separate molecular species in the analyses would reduce intraspecific variation to 0% and 1.3%, respectively. However, it is problematic to use only these two molecular species for the analysis of the 454 field data because of the presence of additional, intermediate lineages in the *Rh. irregularis* group, for which no adequate sampling of sequences is available. The BEG141 clade only accounted for very minor proportions of the *Rh. irregularis s.l.* group in the field, with the only exception of one plot of the no-till treatment (BEG141 2.1%, other *Rh. irregularis* 7% of sequences). We therefore conducted the analyses of the 454 data from the field mainly using the broad definition, well aware that *Rh. irregularis s. l.* may comprise additional taxa which await further characterization.

The barcode gap of RPB1 allowed the separation of almost all morphospecies tested. Exceptions were expected for species in the genus *Gigaspora*, which also show extremely similar nuclear rDNA sequences [47]. Borriello et al. [48] recently demonstrated that this is equally the case for the mitochondrially-encoded COI gene. It is well established that this taxon complex needs more thorough investigation, because these morphospecies are neither resolved with ITS nor with SSU and LSU [47]. Some authors have in fact suggested that some *Gigaspora* spp. might be conspecific, which would explain this finding (J. Morton pers. comm.).

An additional advantage of using a protein-coding gene lies in the use of the respective amino acid sequence as a backbone for error correction of the pyrosequencing data [31], [34], [49]. If these errors are not corrected, diversity will be overestimated [50], [51]. The exons of the RPB1 gene are perfectly suitable for this error correction method and will provide an advantage over the rRNA gene region currently used for 454 AMF community analyses. Although the use of secondary structures of the rRNA genes as a guide for alignment is also possible [52], amino acid sequences can be more easily applied as guide for error corrections and alignments. Therefore it would be a great advantage to incorporate alignment tools such as MACSE, into the advanced pipelines for high-throughput sequencing analysis.

Initial community analysis, based on a 0.8% threshold empirically obtained from the dataset as the minimum distance between morphologically defined species (except *Gigaspora*), revealed 105 OTUs, which would be certainly an overestimation of the total species in the field. After assigning our environmental sequences to known sequences, we obtained 20 assigned species-level MTs. This discrepancy is probably partly due to unequal intraspecific variation among the different clades. More sequences of characterized AMF species are necessary to define either a more suitable cut-off or use the species assignment method of RAxML and QIIME more efficiently but finding a single valid cutoff value for all lineages of *Glomeromycota* may still be elusive.

Based on analyses of spores from the field and from trap cultures, Jansa *et al.* [12] found a total of 18 morphologically-defined species in the same field site we studied here. Our richness estimate of 20 molecular species is in the same range but it is not possible to link all morphology-based taxa of those authors to our molecular data, because of either the absence of RPB1 sequences of these species, or even (in many cases) the non-availability of any molecular sequence. Generally, as demonstrated recently by Borriello *et al.* [16], discrepancies are to be expected when comparing spore- and root-based approaches.

We could clearly relate three out of our seven molecularly identified species to the study of Jansa *et al.* [12] namely *Fu. mosseae (Glomus mosseae)*, *Fu. caledonius (Glomus caledonium)*, and *Rhizophagus irregularis (Glomus intraradices)*. We also detected fungi in the *Cl. claroideum/etunicatum* group, which may correspond to the *Glomus claroideum* spores reported by Jansa *et al.* [12]. Thonar *et al.* [53] also detected *Gi. margarita* and *Ce. pellucida (Scutellospora pellucida)*. Our analysis revealed some closely related species, and we could not confirm that the species we found (*Gigaspora* sp. and *Cetraspora nodosa*) are either conspecific or different to theirs, but it is interesting to note that *Ce. pellucida* (BEG153) clusters together with *Ce. nodosa* (BEG4) in a phylogenetic analysis based on rDNA [47].

On the other hand, a substantial proportion of the sequences we found belonged to taxa that clearly went unnoticed in the Jansa *et al.* [12], [13] studies, such as *Diversispora*, *Archaeospora*, *Ambispora* and *Paraglomus* lineages, some of which might have gone undetected due to their small spore size and low prevalence. Interestingly, the latter three genera are normally also not detected in many molecular approaches due to primer mismatches [44]. The presence of *Archaeospora* and *Ambispora* in arable soils based on molecular detection has only rarely been reported [16], [54], whereas *Paraglomus* has already been recognized as being widespread in this type of environment [55].

Dominant MT in the different treatments and their quantitative relations to a large extent correspond well to those highlighted by Thonar *et al.* [53], but it should be kept in mind that sampling campaigns of the two studies were six years apart and we already found considerable differences among the plots (Figure 5). In the tillage treatment, the predominant AMF was *Fu. mosseae*, this is in agreement with the notion of this species being a disturbance colonizer [56] strongly favoured by agriculture [57]. Nevertheless, *Fu. mosseae* spores were more abundant in fallow field in comparison to a tilled field [58]. The most abundant MT in the chiselled plots (and the second most abundant in the tilled ones) was *Rh. irregularis*. Its relatively low occurrence in the no-till treatments supports the hypothesis put forward by Börstler *et al.* [14] that the genetic diversity of its populations is positively correlated with its abundance. These authors found the lowest population diversity of *Rh. irregularis* in the non-till treatment, including some genotypes typically found in grasslands. Additionally, our analysis confirms the finding of Thonar *et al.* [53] of a high incidence of *Gigasporaceae* species in the non-till treatments. However, the dominant symbiont in the no-till treatment was an unknown *Glomeraceae* species in this study. This fungus was not analyzed by Thonar *et al.* [53] because of the unavailability of molecular tools targeting it. Interestingly, Borriello *et al.* [16] found *Gigaspora* species to be equally present in tilled and in non-tilled treatments, but only in soil and never in roots.

The use of the relative numbers of 454 sequences as a measure for the abundance of taxa in pyrosequencing studies has been disputed, because of possible biases in the method, involving PCR or equimolar mixing [59], [60]. The present study offers the particular situation that some species in the same study have been quantified by qPCR [53] and that these results are in very good agreement with our 454 results (Figure S9). We feel therefore that it is justified to carefully discuss the relative amount of sequences obtained from each of the different taxa as an approximate indication of taxon abundance. Of course possible primer biases would have to be taken into account [61], but as a single copy gene, using RPB1 in semi-quantitative or quantitative approaches would help to avoid pitfalls of copy number polymorphism which was reported even among isolates of a single AMF species [62].

Kohout et al. [61] recently compared the different community compositions obtained by using rDNA primers and primer sets and concluded that the broadest taxon sample was usually obtained using primer sets and that the different systems resulted in different biases of the community.

It is interesting to note that there were few clear-cut qualitative differences among the treatments: the presence in the tillage treatment of seven out of the eight currently recognized AMF families for which RPB1 data are available, is remarkable. This paucity of clear qualitative patterns highlights the need for quantitative approaches to pick up relevant effects of soil management, such as the qPCR approach employed by Thonar *et al.* [53]. However, taxa which are not known *a priori* are impossible to study by qPCR, thus our non-targeted pyrosequencing approach may represent a valid compromise, as it apparently also mirrors correctly major quantitative trends revealed by qPCR. The depth of analysis possible by pyrosequencing allows to show that many AMF MTs survive at low frequencies even under adverse conditions, but also that some of them become virtually extinct by inappropriate management, even in a relatively small-scaled system such as the one studied here.

In this study, we were able to observe in PCAs of Unifrac distance a separation between disturbance and low disturbance treatments, and even some smaller differences among the three treatments. Although the usage of UniFrac distance metric is still debated [63], [64], the statistical analyses with ANOSIM show the same tendencies.

In summary, RPB1 proved to be a valuable tool to study AMF communities under field conditions which is highly likely to facilitate community analyses and contribute to a better understanding of the field ecology of this symbiosis.

## Supporting Information

Data File S1 **All commands used during QIIME analysis.**
(TXT)

Figure S1 **Overview of tong-term tillage experimental field site in Tänikon (Switzerland).**
(PDF)

Figure S2 **Amplification overview of the 454 experiment.**
(PDF)

Figure S3 **Schematic workflow of the 454 experiment.**
(PDF)

Figure S4 **EPA-RAxML tree showing OTUs and reference sequences. OTU's are labeled according to the original QIIME taxonomic assignment.**
(PDF)

Figure S5 **Reference sequences and environmental sequences representative for OTUs were combined to calculate a phylogenetic tree using Neighbor-Joining** [1] **and the Kimura 2- parameter method** [3] **in MEGA5** [4] **.** Bootstrap values from 1000 replicates are shown next to the branches, if over 50% [2]. The analysis involved 198 nucleotide sequences. All ambiguous positions were removed for each sequence pair. There were a total of 462 positions in the final dataset. OTUs are labeled according to the original QIIME taxonomic assignment.
(PDF)

Figure S6 **Barcode gap analysis of the RPB1 fragment RPB1-160f to RPB1-DR1210r, based on K2P distances.** Every sequence was plotted with its corresponding intraspecific variation and the minimum interspecific variation of this sequence. Bars indicate K2P difference between the maximum intraspecific (bottom) and minimum interspecific divergence (top) of each sequence. The red asterisk indicates sequences with small K2P distances of the three species *Gigaspora rosea*, *Gi. gigantea* and *Gi. candida*, which are not resolved. Two of these species are assumed to be conspecific. Colored bars represent sequences of the following species: apricot - *Funneliformis mosseae*, magenta – *Rhizophagus irregularis*, green – *Rhizophagus clarus*, red – *Ambispora leptoticha*. Bars touching zero at their lower end stand for species represented by a single sequence.
(PDF)

Figure S7 **Rarefaction curves based on number of OTUs for the three treatments: no tillage (blue), tillage (orange) and chisel (red).**
(TIF)

Figure S8 **Principal Coordinates Analysis of unweighted UniFrac distances.**
(TIF)

Figure S9 **Taxon distribution summarized by treatment.**
(PDF)

Table S1 **Primers and PCR conditions used for the reference datasets.**
(XLSX)

Table S2 **Details for all Primers used in the 454 studies.**
(XLSX)

Table S3 **OTUs found in the different samples, with their taxonomic assignment.**
(XLSX)

## References

[1] Schüßler A, Walker C (2010) A species list with new families and new genera. ISBN: 978- 1466388048. Available: http://www.amf-phylogeny.com.

[2] Redecker D, Schüßler A, Stockinger H, Stürmer S, Morton J, et al (2013) An evidence-based consensus for the classification of arbuscular mycorrhizal fungi (*Glomeromycota*). Mycorrhiza 23: 515–531. 23558516 10.1007/s00572-013-0486-y

[3] Smith SE, Read DJ (2008) Mycorrhizal symbiosis. Amsterdam; Boston: Academic Press. 800 p.

[4] Walker C, Vestberg M, Demircik F, Stockinger H, Saito M, et al (2007) Molecular phylogeny and new taxa in the *Archaeosporales* (*Glomeromycota*): *Ambispora fennica* gen. sp. nov., *Ambisporaceae* fam. nov., and emendation of *Archaeospora* and *Archaeosporaceae* . Mycol Res 111: 137–153. 17324754 10.1016/j.mycres.2006.11.008

[5] Helgason T, Daniell TJ, Husband R, Fitter AH, Young JPW (1998) Ploughing up the wood-wide web? Nature 394: 431. 9697763 10.1038/28764

[6] Lumini E, Orgiazzi A, Borriello R, Bonfante P, Bianciotto V (2010) Disclosing arbuscular mycorrhizal fungal biodiversity in soil through a land-use gradient using a pyrosequencing approach. Environ Microbiol 12: 2165–2179. 21966911 10.1111/j.1462-2920.2009.02099.x

[7] Hempel S, Renker C, Buscot F (2007) Differences in the species composition of arbuscular mycorrhizal fungi in spore, root and soil communities in a grassland ecosystem. Environ Microbiol 9: 1930–1938. 17635540 10.1111/j.1462-2920.2007.01309.x

[8] Öpik M, Metsis M, Daniell TJ, Zobel M, Moora M (2009) Large-scale parallel 454 sequencing reveals host ecological group specificity of arbuscular mycorrhizal fungi in a boreonemoral forest. New Phytol 184: 424–437. 19558424 10.1111/j.1469-8137.2009.02920.x

[9] James TY, Kauff F, Schoch CL, Matheny PB, Hofstetter V, et al (2006) Reconstructing the early evolution of Fungi using a six-gene phylogeny. Nature 443: 818–822. 17051209 10.1038/nature05110

[10] Schoch CL, Sung GH, López-Giráldez F, Townsend JP, Miadlikowska J, et al (2009) The Ascomycota tree of life: a phylum-wide phylogeny clarifies the origin and evolution of fundamental reproductive and ecological traits. Syst Biol 58: 224–39. 20525580 10.1093/sysbio/syp020

[11] Tisserant E, Malbreil M, Kuo A, Kohler A, Symeonidi A, et al (2013) Genome of an arbuscular mycorrhizal fungus provides insight into the oldest plant symbiosis. Proc Natl Acad Sci 110: 20117–20122. 24277808 10.1073/pnas.1313452110 PMC3864322

[12] Jansa J, Mozafar A, Anken T, Ruh R, Sanders IR, et al (2002) Diversity and structure of AMF communities as affected by tillage in a temperate soil. Mycorrhiza 12: 225–234. 12375133 10.1007/s00572-002-0163-z

[13] Jansa J, Mozafar A, Kuhn G, Anken T, Ruh R, et al (2003) Soil tillage affects the community structure of mycorrhizal fungi in maize roots. Ecol Appl 13

[14] Börstler B, Thiéry O, Sýkorová Z, Berner A, Redecker D (2010) Diversity of mitochondrial large subunit rDNA haplotypes of *Glomus intraradices* in two agricultural field experiments and two semi-natural grasslands. Mol Ecol 19: 1497–1511. 20456234 10.1111/j.1365-294X.2010.04590.x

[15] Verbruggen E, Kiers ET (2010) Evolutionary ecology of mycorrhizal functional diversity in agricultural systems. Evol Appl 3: 547–560. 25567946 10.1111/j.1752-4571.2010.00145.x PMC3352509

[16] Borriello R, Lumini E, Girlanda M, Bonfante P, Bianciotto V (2012) Effects of different management practices on arbuscular mycorrhizal fungal diversity in maize fields by a molecular approach. Biol Fertil Soils 48: 911–922.

[17] Alguacil MM, Lumini E, Roldán A, Salinas-García JR, Bonfante P, et al (2008) The impact of tillage practices on arbuscular mycorrhizal fungal diversity in subtropical crops. Ecol Appl 18: 527–536. 18488613 10.1890/07-0521.1

[18] Naumann M, Schüßler A, Bonfante P (2010) The obligate endobacteria of arbuscular mycorrhizal fungi are ancient heritable components related to the Mollicutes. The ISME Journal 4: 862–71. 20237515 10.1038/ismej.2010.21

[19] Bonfield JK, Smith KF, Staden R (1995) A new DNA sequence assembly program. Nucleic Acids Res 23: 4992–4999. 8559656 10.1093/nar/23.24.4992 PMC307504

[20] Ludwig W, Strunk O, Westram R, Richter L, Meier H, et al (2004) ARB: a software environment for sequence data. Nucleic Acids Res 32: 1363–1371. 14985472 10.1093/nar/gkh293 PMC390282

[21] Stamatakis A (2006) RAxML-VI-HPC: maximum likelihood-based phylogenetic analyses with thousands of taxa and mixed models. Bioinformatics 22: 2688–2690. 16928733 10.1093/bioinformatics/btl446

[22] Tamura K, Peterson D, Peterson N, Stecher G, Nei M, et al (2011) MEGA5: molecular evolutionary genetics analysis using maximum likelihood, evolutionary distance, and maximum parsimony methods. Mol Biol Evol 28: 2731–2739. 21546353 10.1093/molbev/msr121 PMC3203626

[23] Ronquist F, Huelsenbeck JP (2003) MrBayes 3: Bayesian phylogenetic inference under mixed models. Bioinformatics 19: 1572–1574. 12912839 10.1093/bioinformatics/btg180

[24] Brown SDJ, Collins RA, Boyer S, Lefort M, Malumbres-Olarte J, et al (2012) Spider: An R package for the analysis of species identity and evolution, with particular reference to DNA barcoding. Mol Ecol Resour 12: 562–5. 22243808 10.1111/j.1755-0998.2011.03108.x

[25] CRAN (2012) The Comprehensive R Archive Network. Available: http://cran.r-project.org/. Accessed 2012 Apr 9.

[26] Berger SA, Krompass D, Stamatakis A (2011) Performance, accuracy, and Web server for evolutionary placement of short sequence reads under maximum likelihood. Syst Biol 60: 291–302. 21436105 10.1093/sysbio/syr010 PMC3078422

[27] Anken T, Heusser J, Weisskopf P, Zihlmann U, Forrer HR, et al (1997) Agroscope - Bodenbearbeitungssysteme. Direktsaat stellt höchste Anforderungen. Berichte: Swiss Federal Research Station for Agriculture Economics and Engineering 501: 1–14.

[28] Berry D, Ben Mahfoudh K, Wagner M, Loy A (2011) Barcoded primers used in multiplex amplicon pyrosequencing bias amplification. Appl Environ Microbiol 77: 7846–9. 21890669 10.1128/AEM.05220-11 PMC3209180

[29] Frank DN (2009) BARCRAWL and BARTAB: software tools for the design and implementation of barcoded primers for highly multiplexed DNA sequencing. BMC Bioinformatics 10: 362. 19874596 10.1186/1471-2105-10-362 PMC2777893

[30] Caporaso JG, Kuczynski J, Stombaugh J, Bittinger K, Bushman FD, et al (2010) QIIME allows analysis of high-throughput community sequencing data. Nat Methods 7: 335–336. 20383131 10.1038/nmeth.f.303 PMC3156573

[31] Yu DW, Ji Y, Emerson BC, Wang X, Ye C, et al (2012) Biodiversity soup: metabarcoding of arthropods for rapid biodiversity assessment and biomonitoring. Methods Ecol Evol 3: 613–623.

[32] Edgar RC (2010) Search and clustering orders of magnitude faster than BLAST. Bioinformatics 26: 2460–2461. 20709691 10.1093/bioinformatics/btq461

[33] Edgar RC, Haas BJ, Clemente JC, Quince C, Knight R (2011) UCHIME improves sensitivity and speed of chimera detection. Bioinformatics 27: 2194–2200. 21700674 10.1093/bioinformatics/btr381 PMC3150044

[34] Ranwez V, Harispe S, Delsuc F, Douzery EJP (2011) MACSE: Multiple Alignment of Coding SEquences accounting for frameshifts and stop codons. PLoS One 6: e22594. 21949676 10.1371/journal.pone.0022594 PMC3174933

[35] Hao X, Jiang R, Chen T (2011) Clustering 16S rRNA for OTU prediction: a method of unsupervised Bayesian clustering. Bioinformatics 27: 611–8. 21233169 10.1093/bioinformatics/btq725 PMC3042185

[36] Lozupone CA, Hamady M, Kelley ST, Knight R (2007) Quantitative and qualitative beta diversity measures lead to different insights into factors that structure microbial communities. Appl Environ Microbiol 73: 1576–1585. 17220268 10.1128/AEM.01996-06 PMC1828774

[37] Schloss PD, Westcott SL, Ryabin T, Hall JR, Hartmann M, et al (2009) Introducing mothur: open-source, platform-independent, community-supported software for describing and comparing microbial communities. Appl Environ Microbiol 75: 7537–41. 19801464 10.1128/AEM.01541-09 PMC2786419

[38] Redecker D, Raab PA (2006) Phylogeny of the *Glomeromycota* (arbuscular mycorrhizal fungi): recent developments and new gene markers. Mycologia 98: 885–895. 17486965 10.3852/mycologia.98.6.885

[39] Schoch CL, Seifert KA, Huhndorf S, Robert V, Spouge JL, et al (2012) Nuclear ribosomal internal transcribed spacer (ITS) region as a universal DNA barcode marker for Fungi. Proc Natl Acad Sci USA 109: 6241–6246. 22454494 10.1073/pnas.1117018109 PMC3341068

[40] Lin X, Feng Y, Zhang H, Chen R, Wang J, et al (2012) Long-Term balanced fertilization decreases arbuscular mycorrhizal fungal diversity in an arable soil in North China revealed by 454 pyrosequencing. Environ Sci Technol 46: 5764–5771. 22582875 10.1021/es3001695

[41] Stockinger H, Walker C, Schüßler A (2009) ‘*Glomus intraradices* DAOM197198’, a model fungus in arbuscular mycorrhiza research, is not *Glomus intraradices* . New Phytol 183: 1176–1187. 19496945 10.1111/j.1469-8137.2009.02874.x

[42] Nilsson RH, Kristiansson E, Ryberg M, Hallenberg N, Larsson KH (2008) Intraspecific ITS variability in the kingdom fungi as expressed in the international sequence databases and its implications for molecular species Identification. Evol Bioinform Online 4: 193–201. 19204817 10.4137/ebo.s653 PMC2614188

[43] VanKuren NW, den Bakker HC, Morton JB, Pawlowska TE (2013) Ribosomal RNA gene diversity, effective population size, and evolutionary longevity in asexual *Glomeromycota* . Evolution 67: 207–224. 23289573 10.1111/j.1558-5646.2012.01747.x

[44] Stockinger H, Krüger M, Schüßler A (2010) DNA barcoding of arbuscular mycorrhizal fungi. New Phytol 187: 461–474. 20456046 10.1111/j.1469-8137.2010.03262.x

[45] Croll D, Wille L, Gamper HA, Mathimaran N, Lammers PJ, et al (2008) Genetic diversity and host plant preferences revealed by simple sequence repeat and mitochondrial markers in a population of the arbuscular mycorrhizal fungus *Glomus intraradices* . New Phytol 178: 672–687. 18298433 10.1111/j.1469-8137.2008.02381.x

[46] Croll D, Corradi N, Gamper HA, Sanders IR (2008) Multilocus genotyping of arbuscular mycorrhizal fungi and marker suitability for population genetics. New Phytol 180: 564–568. 18684159 10.1111/j.1469-8137.2008.02602.x

[47] Krüger M, Krüger C, Walker C, Stockinger H, Schüßler A (2012) Phylogenetic reference data for systematics and phylotaxonomy of arbuscular mycorrhizal fungi from phylum to species level. New Phytol 193: 970–984. 22150759 10.1111/j.1469-8137.2011.03962.x

[48] Borriello R, Bianciotto V, Orgiazzi A, Lumini E, Bergero R (2014) Sequencing and comparison of the mitochondrial COI gene from isolates of arbuscular mycorrhizal fungi belonging to *Gigasporaceae* and *Glomeraceae* families,. Mol Phylogenet Evol 75: 1–10. 24569015 10.1016/j.ympev.2014.02.012

[49] Emerson BC, Cicconardi F, Fanciulli PP, Shaw PJA (2011) Phylogeny, phylogeography, phylobetadiversity and the molecular analysis of biological communities. Philos Trans R Soc Lond B Biol Sci 366: 2391–2402. 21768154 10.1098/rstb.2011.0057 PMC3130430

[50] Quince C, Lanzén A, Curtis TP, Davenport RJ, Hall N, et al (2009) Accurate determination of microbial diversity from 454 pyrosequencing data. Nat Methods 6: 639–641. 19668203 10.1038/nmeth.1361

[51] Reeder J, Knight R (2010) Rapidly denoising pyrosequencing amplicon reads by exploiting rank-abundance distributions. Nat Methods 7: 668–669. 20805793 10.1038/nmeth0910-668b PMC2945879

[52] Nawrocki EP, Kolbe DL, Eddy SR (2009) Infernal 1.0: inference of RNA alignments. Bioinformatics 25: 1335–1337. 19307242 10.1093/bioinformatics/btp157 PMC2732312

[53] Thonar C, Erb A, Jansa J (2012) Real-time PCR to quantify composition of arbuscularmycorrhizal fungal communities—marker design, verification, calibration and field validation. Mol Ecol Resour 12: 219–232. 22059700 10.1111/j.1755-0998.2011.03086.x

[54] Brito I, Goss MJ, de Carvalho M, Chatagnier O, van Tuinen D (2012) Impact of tillage system on arbuscular mycorrhiza fungal communities in the soil under Mediterranean conditions. Soil and Tillage Research 121: 63–67.

[55] Hijri I, Sýkorová Z, Oehl F, Ineichen K, Mäder P, et al (2006) Communities of arbuscular mycorrhizal fungi in arable soils are not necessarily low in diversity. Mol Ecol 15: 2277–2289. 16780440 10.1111/j.1365-294X.2006.02921.x

[56] Sýkorová Z, Ineichen K, Wiemken A, Redecker D (2007) The cultivation bias: different communities of arbuscular mycorrhizal fungi detected in roots from the field, from bait plants transplanted to the field, and from a greenhouse trap experiment. Mycorrhiza 18: 1–14. 17879101 10.1007/s00572-007-0147-0

[57] Rosendahl S, McGee P, Morton JB (2009) Lack of global population genetic differentiation in the arbuscular mycorrhizal fungus *Glomus mosseae* suggests a recent range expansion which may have coincided with the spread of agriculture. Mol Ecol 18: 4316–4329. 19765226 10.1111/j.1365-294X.2009.04359.x

[58] Rosendahl S, Matzen HB (2008) Genetic structure of arbuscular mycorrhizal populations in fallow and cultivated soils. New Phytol 179: 1154–1161. 18565143 10.1111/j.1469-8137.2008.02535.x

[59] Harris JK, Sahl JW, Castoe TA, Wagner BD, Pollock DD, et al (2010) Comparison of normalization methods for construction of large, multiplex amplicon pools for next-generation sequencing. Appl Environ Microbiol 76: 3863–3868. 20418443 10.1128/AEM.02585-09 PMC2893486

[60] Amend AS, Seifert KA, Bruns TD (2010) Quantifying microbial communities with 454 pyrosequencing: does read abundance count? Mol Ecol 19: 5555–5565. 21050295 10.1111/j.1365-294X.2010.04898.x

[61] Kohout P, Sudová R, Janoušková M, Čtvrtlíková M, Hejda M, et al (2014) Comparison of commonly used primer sets for evaluating arbuscular mycorrhizal fungal communities: Is there a universal solution? Soil Biol Biochem 68: 482–493.

[62] Corradi N, Croll D, Colard A, Kuhn G, Ehinger M, et al (2007) Gene copy number polymorphisms in an arbuscular mycorrhizal fungal population. Appl Environ Microbiol 73: 366–369. 17085714 10.1128/AEM.01574-06 PMC1797111

[63] Schloss PD (2008) Evaluating different approaches that test whether microbial communities have the same structure. ISME Journal 2: 265–275. 18239608 10.1038/ismej.2008.5

[64] Lozupone C, Lladser ME, Knights D, Stombaugh J, Knight R (2011) UniFrac: an effective distance metric for microbial community comparison. ISME Journal 5: 169–172. 20827291 10.1038/ismej.2010.133 PMC3105689

