# Supplementary material for: The Largest Subunit of RNA Polymerase II as a New Marker Gene to Study Assemblages of Arbuscular Mycorrhizal Fungi in the Field
Source: PLoS One. 2014 Oct 2;9(10):e107783. doi: 10.1371/journal.pone.0107783 (PMC4183475; doi:10.1371/journal.pone.0107783)
Supplement: Figure S1 — Overview of tong-term tillage experimental field site in Tänikon (Switzerland). (PDF) [file pone.0107783.s002.pdf]

Figure S1: Overview of tong-term tillage experimental field site in Tänikon (Switzerland).

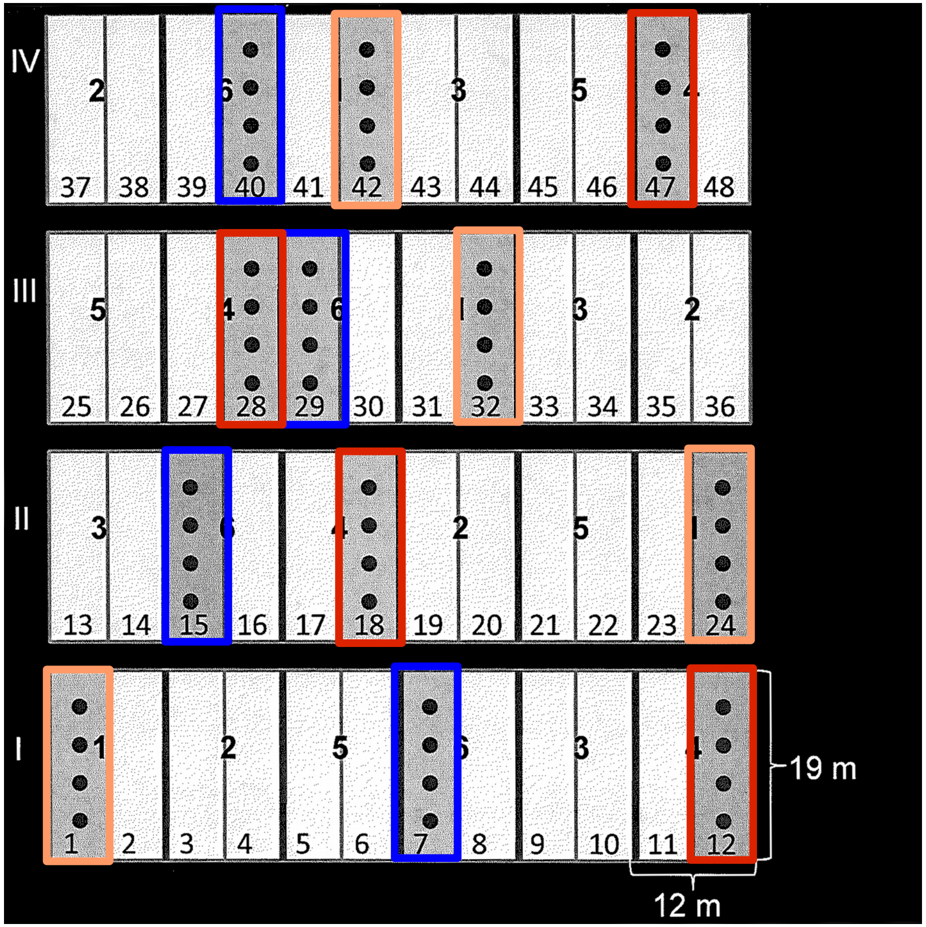

Long-term tillage experiment in Tänikon (Switzerland). Sampling details (Börstler *et al* 2010 and Jansa *et al.* 2002 & 2003)

No Tillage

Chisel

Tillage
