# Supplementary figures and images for: The Largest Subunit of RNA Polymerase II as a New Marker Gene to Study Assemblages of Arbuscular Mycorrhizal Fungi in the Field
Source: PLoS One. 2014 Oct 2;9(10):e107783. doi: 10.1371/journal.pone.0107783 (PMC4183475; doi:10.1371/journal.pone.0107783)

Figure S2: Amplification overview 454 experiment

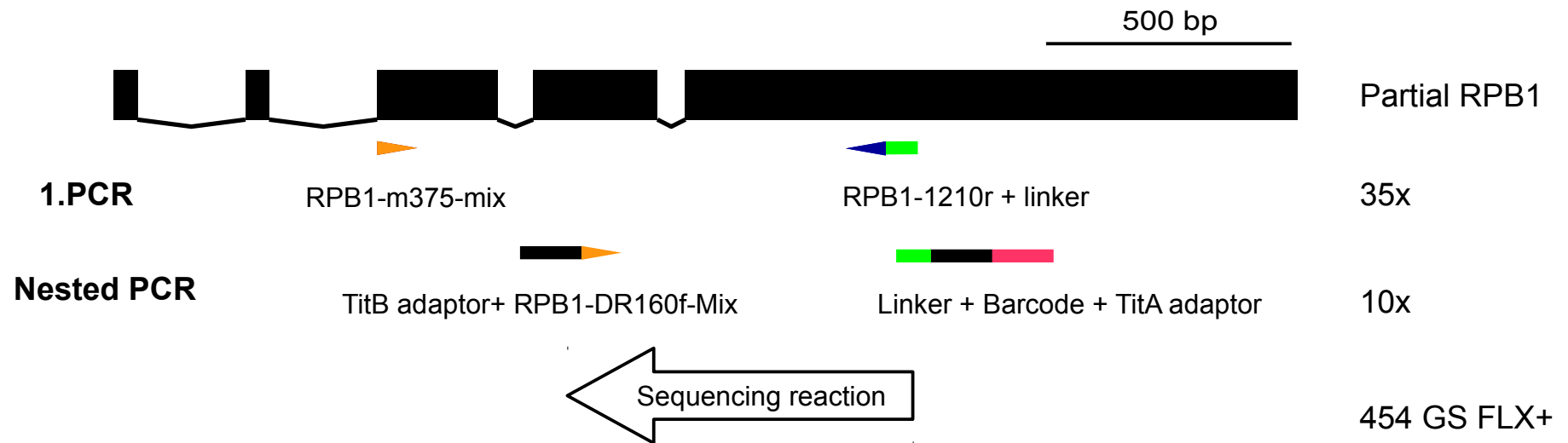

Supplement: Figure S2 — Amplification overview of the 454 experiment. (PDF) [file pone.0107783.s003.pdf]

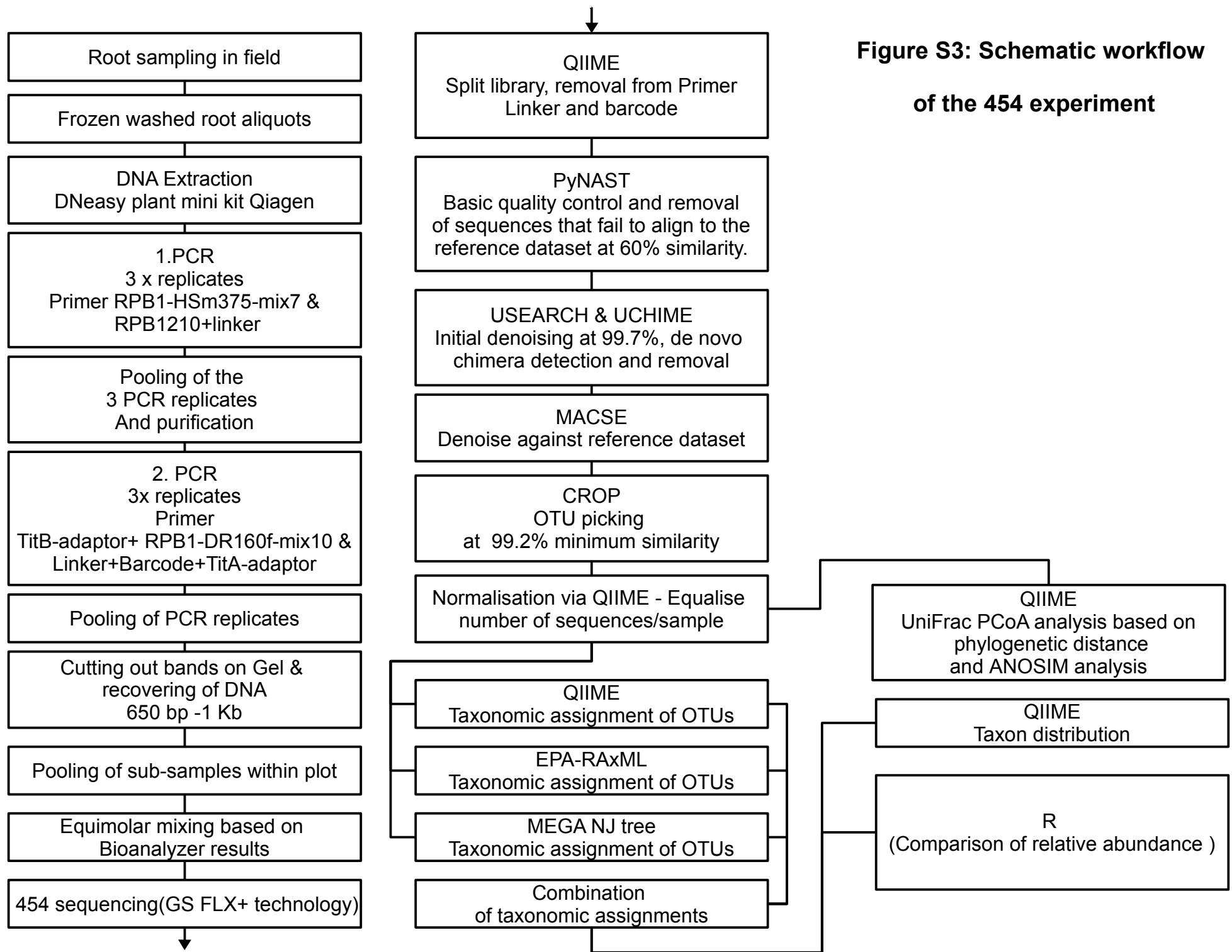

Supplement: Figure S3 — Schematic workflow of the 454 experiment. (PDF) [file pone.0107783.s004.pdf]

**Figure S6: Barcode gap analysis of the RPB1 fragment RPB1-160f to RPB1-DR1210r, based on K2P distances.**

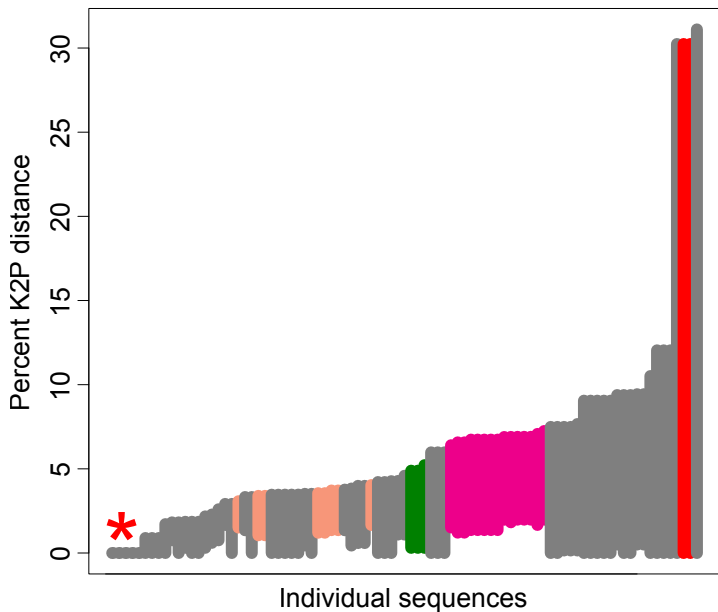

Supplement: Figure S6 — Barcode gap analysis of the RPB1 fragment RPB1-160f to RPB1-DR1210r, based on K2P distances. Every sequence was plotted with its corresponding intraspecific variation and the minimum interspecific variation of this sequence. Bars indicate K2P difference between the maximum intraspecific (bottom) and minimum interspecific divergence (top) of each sequence. The red asterisk indicates sequences with small K2P distances of the three species Gigaspora rosea, Gi. gigantea and Gi. candida, which are not resolved. Two of these species are assumed to be conspecific. Colored bars represent sequences of the following species: apricot - Funneliformis mosseae, magenta – Rhizophagus irregularis, green – Rhizophagus clarus, red – Ambispora leptoticha. Bars touching zero at their lower end stand for species represented by a single sequence. (PDF) [file pone.0107783.s007.pdf]

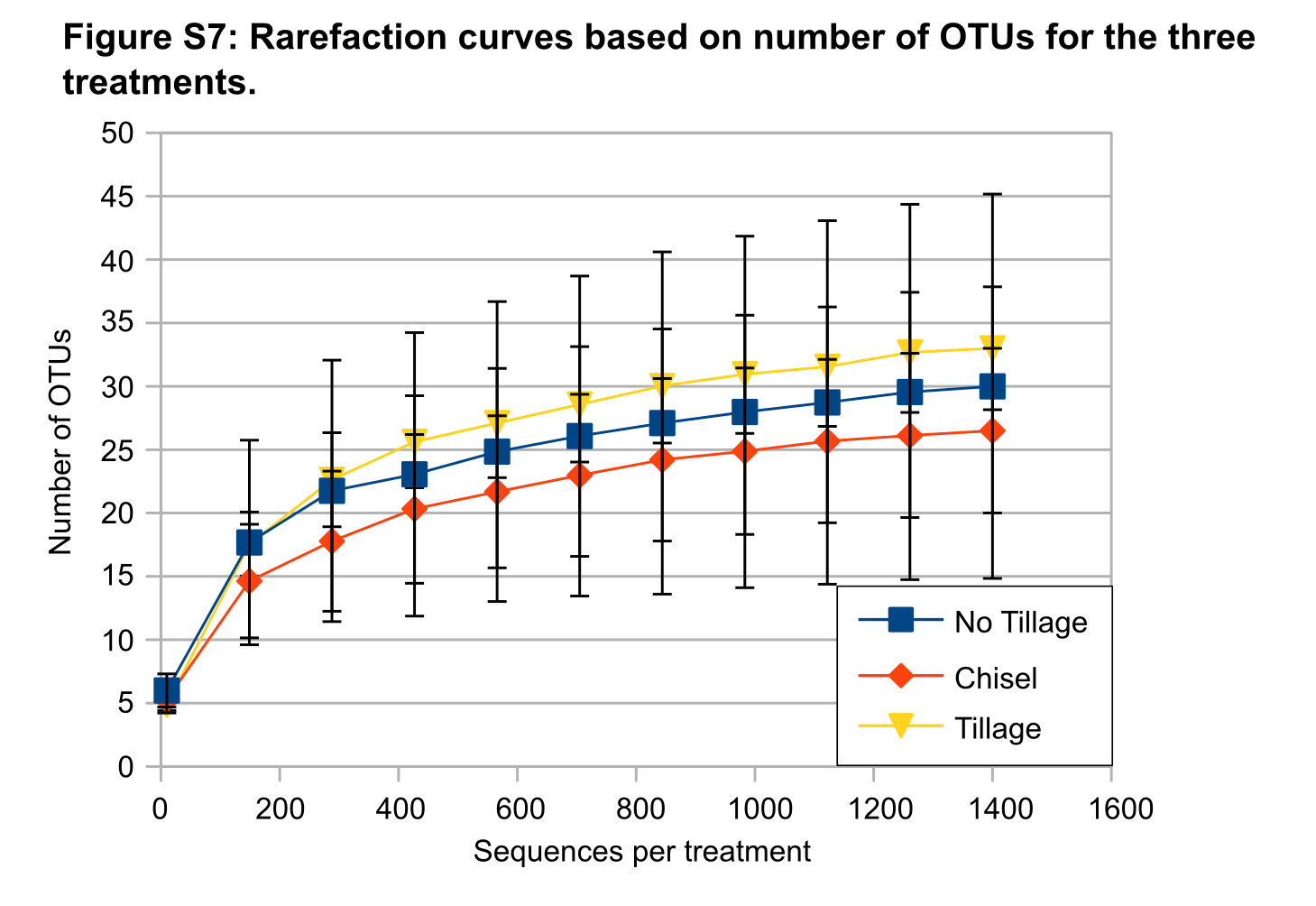

Supplement: Figure S7 — Rarefaction curves based on number of OTUs for the three treatments: no tillage (blue), tillage (orange) and chisel (red). (TIF) [file pone.0107783.s008.tif]

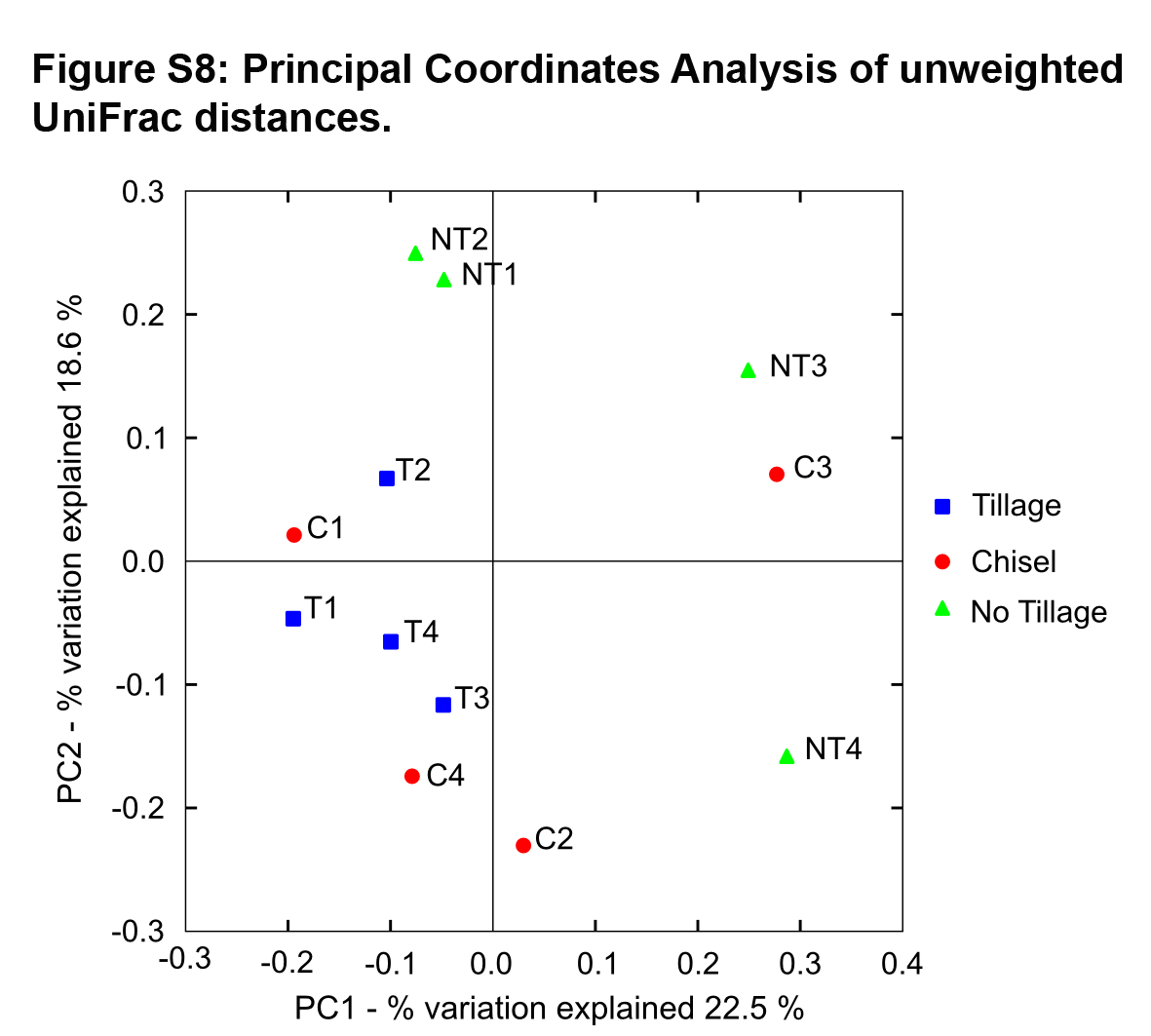

Supplement: Figure S8 — Principal Coordinates Analysis of unweighted UniFrac distances. (TIF) [file pone.0107783.s009.tif]

**Figure S9: Taxon distribution summarized by treatment.**

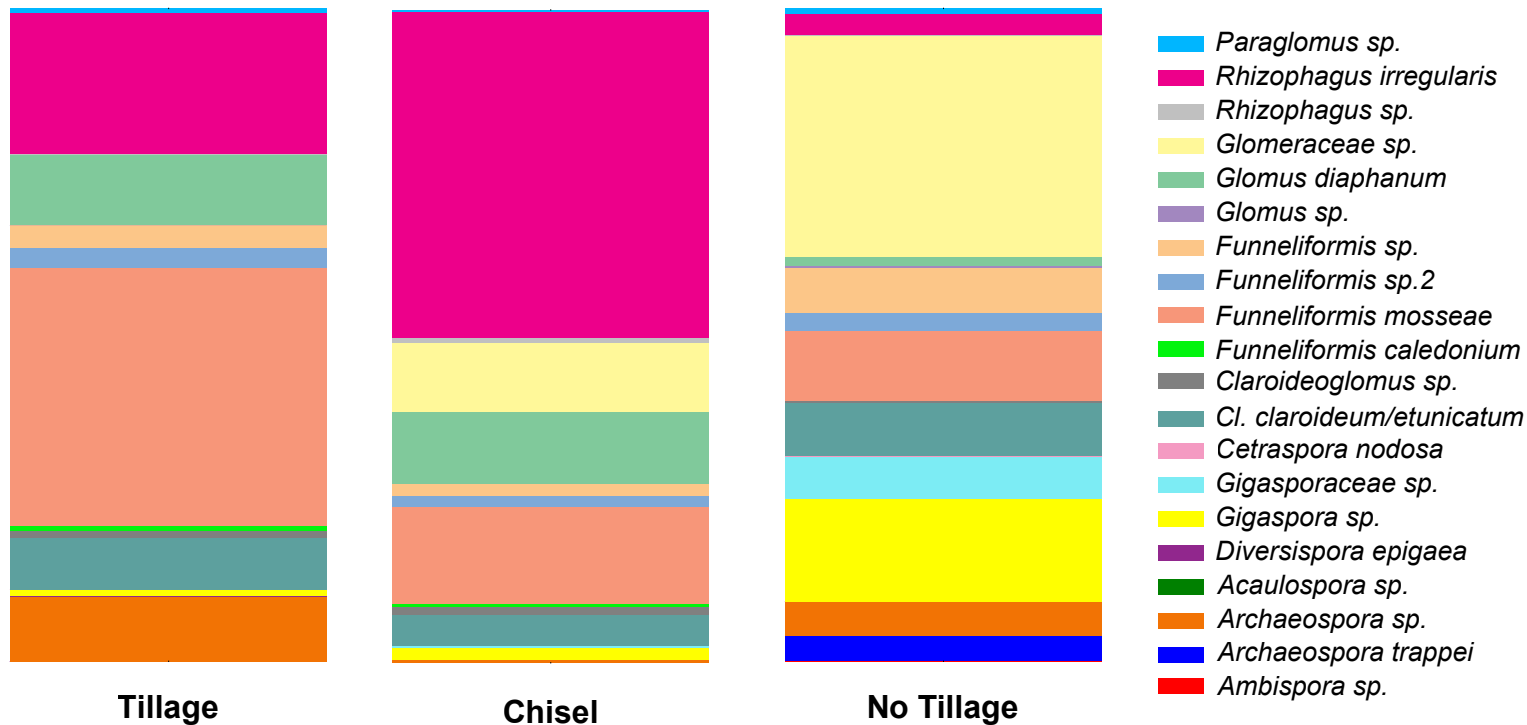

Supplement: Figure S9 — Taxon distribution summarized by treatment. (PDF) [file pone.0107783.s010.pdf]
