# Supplementary material for: The Largest Subunit of RNA Polymerase II as a New Marker Gene to Study Assemblages of Arbuscular Mycorrhizal Fungi in the Field
Source: PLoS One. 2014 Oct 2;9(10):e107783. doi: 10.1371/journal.pone.0107783 (PMC4183475; doi:10.1371/journal.pone.0107783)

Figure S4: EPA-RAxML tree showing OTUs and reference sequences. OTU's are labeled according to the original QIIME taxonomic assignment.

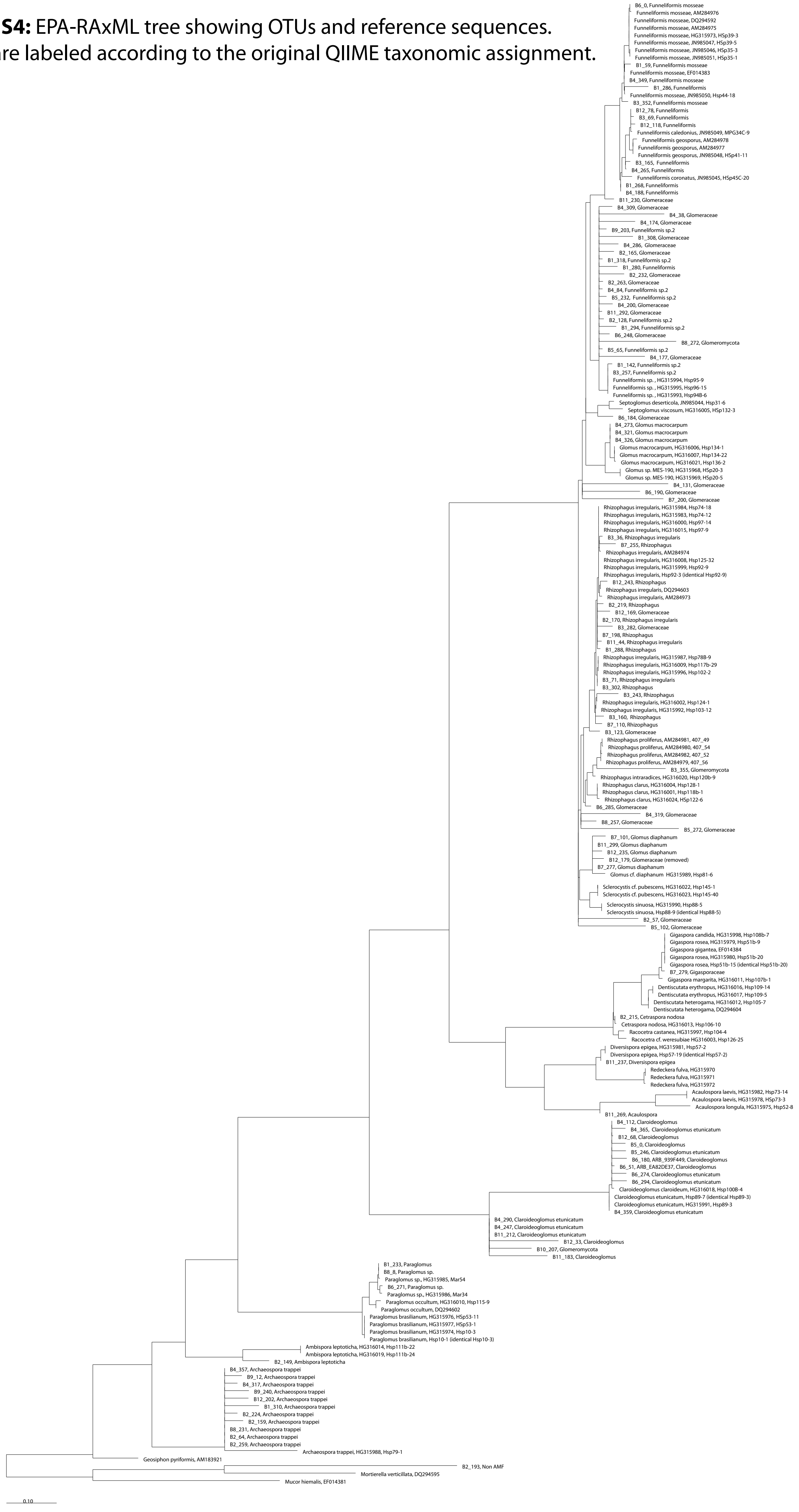

Supplement: Figure S4 — EPA-RAxML tree showing OTUs and reference sequences. OTU's are labeled according to the original QIIME taxonomic assignment. (PDF) [file pone.0107783.s005.pdf]
