# Supplementary material for: The Largest Subunit of RNA Polymerase II as a New Marker Gene to Study Assemblages of Arbuscular Mycorrhizal Fungi in the Field
Source: PLoS One. 2014 Oct 2;9(10):e107783. doi: 10.1371/journal.pone.0107783 (PMC4183475; doi:10.1371/journal.pone.0107783)

Figure S5: Reference sequences and environmental sequences representative for OTUs were combined to calculate a phylogenetic tree using Neighbor-Joining [1] and the Kimura 2- parameter method [3] in MEGA5 [4]. Bootstrap values from 1000 replicates are shown next to the branches, if over 50% [2]. The analysis involved 198 nucleotide sequences. All ambiguous positions were removed for each sequence pair. There were a total of 462 positions in the final dataset. OTUs are labeled according to the original QIIME taxonomic assignment.

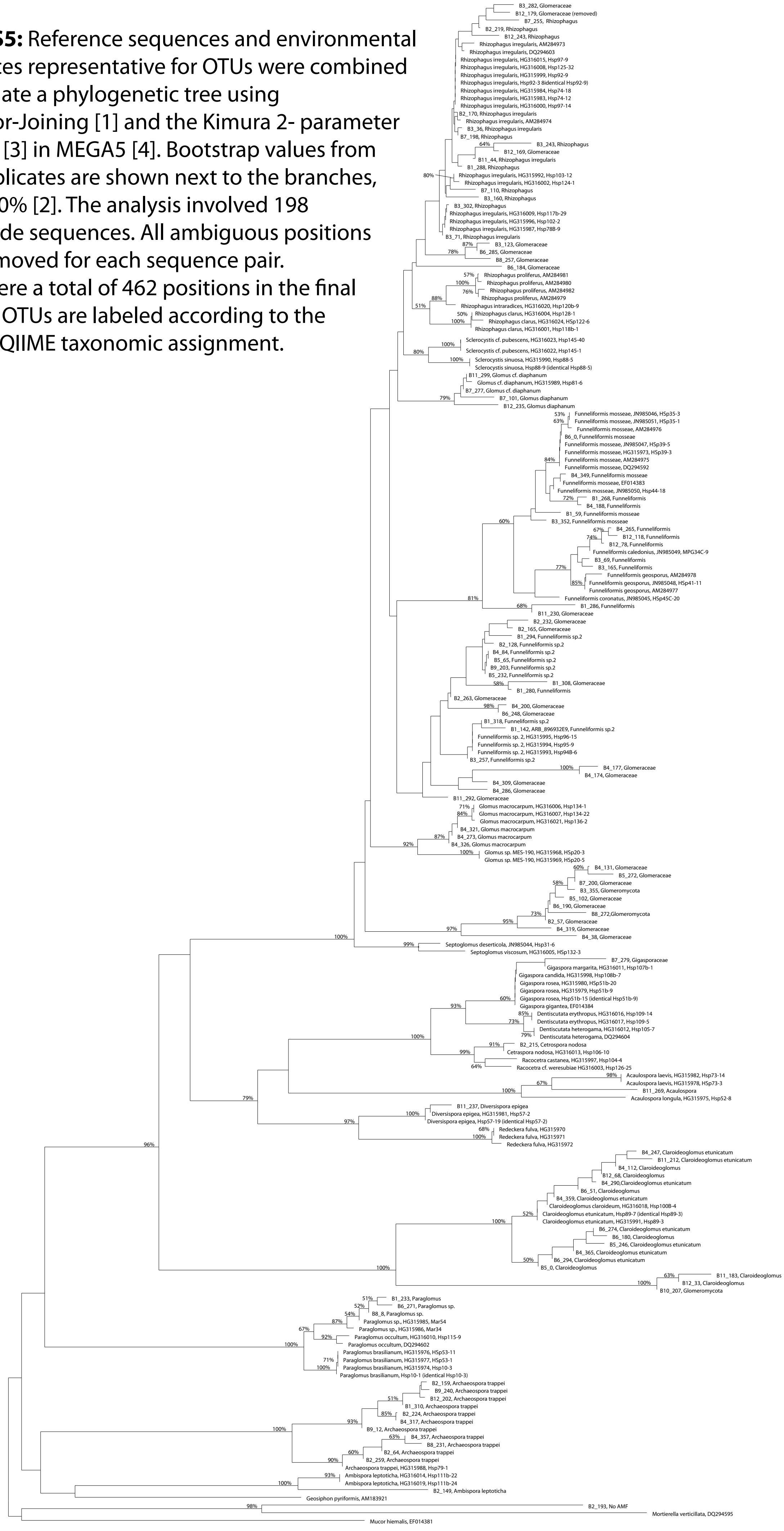

Supplement: Figure S5 — Reference sequences and environmental sequences representative for OTUs were combined to calculate a phylogenetic tree using Neighbor-Joining [1] and the Kimura 2- parameter method [3] in MEGA5 [4] . Bootstrap values from 1000 replicates are shown next to the branches, if over 50% [2]. The analysis involved 198 nucleotide sequences. All ambiguous positions were removed for each sequence pair. There were a total of 462 positions in the final dataset. OTUs are labeled according to the original QIIME taxonomic assignment. (PDF) [file pone.0107783.s006.pdf]
